# Realistic noise-tolerant randomness amplification using finite number of devices

**DOI:** 10.1038/ncomms11345

**Published:** 2016-04-21

**Authors:** Fernando G. S. L. Brandão, Ravishankar Ramanathan, Andrzej Grudka, Karol Horodecki, Michał Horodecki, Paweł Horodecki, Tomasz Szarek, Hanna Wojewódka

**Affiliations:** 1 Quantum Architectures and Computation Group, Microsoft Research, Redmond, Washington 98052, USA; 2 Department of Computer Science, University College London, WC1E 6BT London, UK; 3 Faculty of Mathematics, Physics and Informatics, Institute of Theoretical Physics and Astrophysics and National Quantum Information Centre, University of Gdańsk, 80-309 Gdańsk, Poland; 4 Faculty of Physics, Adam Mickiewicz University, 61-614 Poznań, Poland; 5 Faculty of Mathematics, Physics and Informatics, Institute of Informatics and National Quantum Information Centre, University of Gdańsk, 80-309 Gdańsk, Poland; 6 Faculty of Applied Physics and Mathematics, National Quantum Information Centre, Gdańsk University of Technology, 80-233 Gdańsk, Poland; 7 Faculty of Mathematics, Physics and Informatics, Institute of Mathematics and National Quantum Information Centre, University of Gdańsk, 80-309 Gdańsk, Poland; 8 Faculty of Mathematics, Physics and Informatics, Institute of Theoretical Physics and Astrophysics, Instutute of Mathematics and National Quantum Information Centre, University of Gdańsk, 80-309 Gdańsk, Poland

## Abstract

Randomness is a fundamental concept, with implications from security of modern data systems, to fundamental laws of nature and even the philosophy of science. Randomness is called certified if it describes events that cannot be pre-determined by an external adversary. It is known that weak certified randomness can be amplified to nearly ideal randomness using quantum-mechanical systems. However, so far, it was unclear whether randomness amplification is a realistic task, as the existing proposals either do not tolerate noise or require an unbounded number of different devices. Here we provide an error-tolerant protocol using a finite number of devices for amplifying arbitrary weak randomness into nearly perfect random bits, which are secure against a no-signalling adversary. The correctness of the protocol is assessed by violating a Bell inequality, with the degree of violation determining the noise tolerance threshold. An experimental realization of the protocol is within reach of current technology.

## Abstract (web summary)

Quantum mechanics allows to generate nearly ideal random strings from initially weak random sources, important for security of data systems, but this remains elusive in practice. Here the authors propose a realistic, error-tolerant and secure protocol for randomness amplification of arbitrary bits.

## Graphical abstract

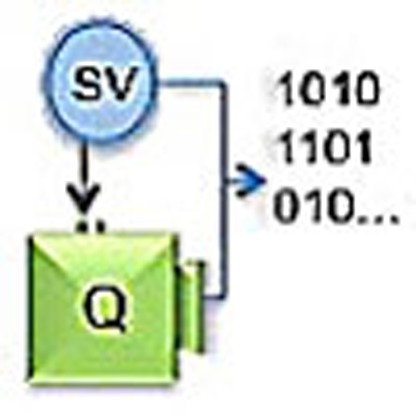

The simplest cryptographic resource is a random bit, unknown to any adversary. It is a basic brick of virtually any cryptographic protocol. However, traditional random number generators (RNGs) are based on classical physics, which is deterministic. Therefore, the output randomness cannot be trusted without further assumptions. For instance, a pseudo-random number generator based on a deterministic algorithm might rely on a small set of initial values (the seed) being unknown to any adversary or on certain mathematical hardness assumptions. Hardware RNGs based on physical phenomena such as thermal or atmospheric noise, radioactive decay or unstable dynamical systems are susceptible to attacks by an adversary feeding known signals into the supposedly random source. Indeed, it is not possible to create randomness out of nothing, and random numbers generated by any sort of software are in principle vulnerable to hacking.

In view of such pervasiveness of determinism, computer scientists have considered the weaker task of amplifying imperfect randomness. The goal here is to extract nearly perfect random bits given many samples of somewhat random, but potentially almost deterministic bits. Unfortunately, even this weaker task was proven to be impossible by Santha and Vazirani in 1984 (ref. 1). They introduced a model of the sources of weak randomness (described in detail later) and showed that randomness amplification from a single such source through any deterministic method is impossible. Classical information processing only allows for randomness amplification when one has access to at least two independent weak sources of randomness.

However, for almost a century, we have been aware that Nature is not ruled by deterministic classical laws. It is described by quantum theory, which is intrinsically non-deterministic, because of the famous rule postulated by Max Born in 1927, stating that the quantum-mechanical wave-function only describes probabilities of events. This peculiar feature of Nature has generated conflict among generations of scientists, but was shown to be an indispensable part of quantum theory by John Bell^2^.

In recent decades, the information revolution has influenced almost every aspect of present-day life and stimulated a huge effort towards establishing cryptographic security. Quantum indeterminism has now been promoted from a weird peculiarity of quantum theory to a potentially important resource. New RNGs, based on quantum principles, have been built and are now commercially available (for example, the ones offered by IdQuantique^3^). However, to trust the randomness produced by such devices, one has to either trust that the device works as per its specification or verify its internal construction by direct inspection. As a typical user would not be able to make such a verification, a basic question arises: can we build a device to produce certified randomness, in a device-independent way?

In view of the requirement of using untrusted devices, we still face the same limitation as in traditional cryptography: randomness cannot be created out of nothing. Fortunately, recently it turned out that randomness amplification, while inaccessible classically, can be accomplished in a device-independent manner by exploiting quantum-mechanical systems^4^. Earlier work has also shown that the similar task of randomness expansion (in which a small fully random seed is stretched into a longer random string) is possible using quantum-mechanical correlations^5^. The central role in both these processes is played by the so-called Bell inequalities^2^. The test of violation of Bell inequalities, which certifies true randomness, is performed solely on the statistics, and therein lies its device-independent potential. This idea first appeared in a seminal paper by Ekert^6^, followed by Barrett *et al*.^7^, as well as others.

There is, however, a caveat to this concept. Namely, there is no way to guarantee the random nature of the world, because of the possibility of super-determinism, that is, all events, including the choices of the measurement settings in any experiment, may be predetermined (no free-will). Indeed, a test of violation of Bell inequalities allows to uncover true randomness only if we can choose the measurement settings at random. We thus face a sort of vicious circle.

Recently, Colbeck and Renner, building on a breakthrough results in refs 5, 6, 7, showed that one can amplify randomness: even though the inputs are only weakly random, the outputs provide almost ideal randomness. Subsequent protocols have further developed this original result^8^,^9^.

The existing protocols that implement the task of amplifying randomness, while ingenious and conceptually important, have some drawbacks that make them impossible to implement in reality. Namely, after each single round of measurement, the device has to be discarded and new devices have to be used (equivalently, a large number of devices may be used in parallel). To imagine how limited such a protocol would be in practice, consider a typical implementation in which we need to create a kilo-byte of secure randomness. Then, even if we used a single photon per output random bit, 8,000 devices, shielded from each other, would be required.

Only one protocol proposed so far does not have this unfeasible requirement of many devices—the original Colbeck and Renner's protocol^4^. However, it also suffers from many disadvantages—namely, the protocol does not tolerate noise, besides using a large number of settings, being therefore impractical. Thus, the existing protocols are trapped between Scylla of many devices and Charybdis of fragility to noise. There have been other proposals to solve this problem in refs 10, 11 for an adversary obeying quantum-mechanical laws. However, full proofs of security of these protocols are still missing.

In contrast, good protocols have been developed for the task of randomness expansion^12^. Therefore, it has been a pressing open question in the field whether randomness amplification can realistically be implemented.

In this paper, we provide protocols that escape this dual restrictive alternative, being therefore directly implementable in practice. We propose protocols that use a small constant number of devices and produce randomness out of an arbitrarily weak SV-source while being able to tolerate a constant noise rate. Moreover, in the protocols the security can be tested without referring to quantum mechanics.

## Results

### Santha–Vazirani *ɛ*-SV sources

As an illustration of the SV-source model, assume that an adversary has two different coins, one biased towards heads (for example, probability of heads is 2/3) and the other biased towards tails (probability of heads is 1/3, say). The adversary, in each time step, chooses one of the two coins and tosses it; the choice of coin may depend (probabilistically) on the outcomes of the prior tosses. The sequence of random outcomes of these coin tosses then gives an SV-source.

In general, Santha and Vazirani^1^ considered an *ɛ*-SV source, where *ɛ*∈[0,0.5] is a parameter, which indicates how much the source bits deviate from fully random (in the above example, *ɛ*=1/6). In the most general case, an *ɛ*-SV source is given by a probability distribution *p*(*ϕ*_0_,…,*ϕ*_*n*_) over bit strings such that


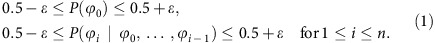


Note that, when *ɛ*=0, the bits are fully random, whereas for *ɛ*=0.5, they are fully deterministic.

The Santha–Vazirani no-go result holds for any *ɛ*≠0 and says that any bit extracted by a deterministic procedure from a single *ɛ*-SV source will always have an *ɛ* bias.

### Bell inequalities

In the Nature, there exist correlations that cannot be described by any deterministic theory, that is, the correlations cannot be explained by any model where all the randomness is due to lack of knowledge. Such correlations are manifested operationally through the violation of Bell inequalities. This immediately brings to mind applications in security: were the probabilistic description simply due to lack of one's knowledge, an eavesdropper could potentially have this knowledge. Thus, the security would be compromised and there would be no randomness whatsoever. On the contrary, violation of Bell inequalities makes room for the possibility for true randomness.

### Main results

We propose here two protocols, which are both discussed in detail, as well as compared with similar protocols for randomness amplification, in Supplementary Note 1. The Bell inequality and randomness extractors used in the protocols are presented in Supplementary Note 2. Assumptions are summarized in Supplementary Note 3, while the main mathematical tools, essential to establish the security of the protocols, are summarized in Supplementary Note 4.

**Figure 1:**
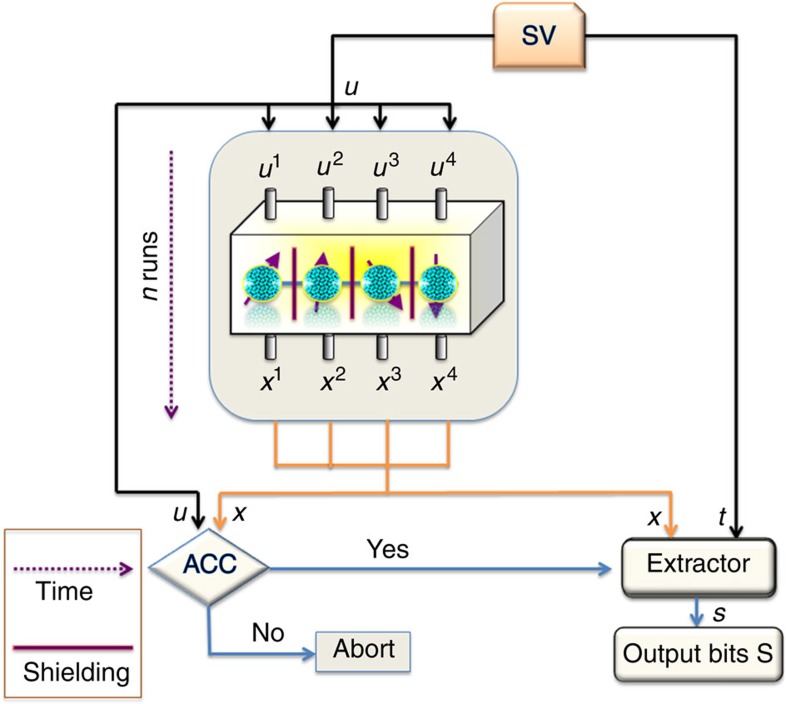
Protocol I—designed for randomness amplification with four devices. Devices are shielded one from another and used in a sequence of *n* runs. ***u***^1^,…***u***^4^ denote binary inputs in each run and they determine which measurement is made in the given run, whereas ***x***^1^,…,***x***^4^ are binary outputs of the measurements. If the test, performed on the inputs and outputs of the device, is passed (denoted by ACC—Yes), then the outputs and another set of bits from the SV-source (denoted by *t*) are introduced into an extractor in order to obtain final output bits *S*. Black arrows mark the directions of bits from the SV-source; orange ones indicate where output bits are introduced and blue arrows show possible alternatives: accepting the protocol and obtaining (supposedly random) bits or aborting the protocol.

**Figure 2:**
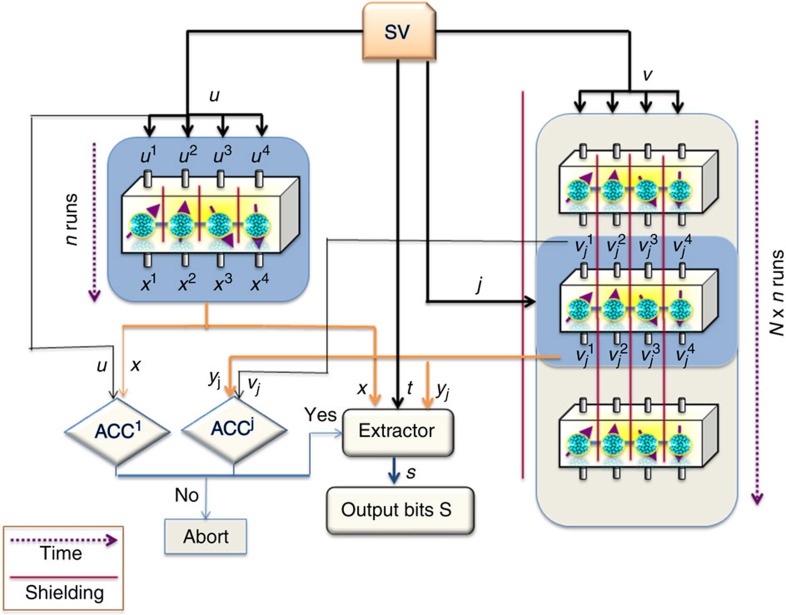
Protocol II—designed for randomness amplification from eight devices. The eight devices are shielded from each other and one block of *n* runs is performed sequentially on the first four devices while *N* blocks of *n* runs are performed sequentially on the second four devices. One of these *N* blocks, marked in blue, is selected using some portion of bits (denoted by *j*) from the SV-source. 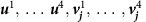 denote binary inputs in each run and they determine which measurement is made in the given run, whereas 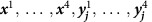 are binary outputs of the measurements. Black arrows indicate where bits from SV source are directed, whereas orange ones mark where output bits are introduced. The test is performed separately on inputs and outputs of the first four devices and the chosen block from the second four devices. Blue arrows show possible alternatives: either the tests are passed (ACC^1^—Yes, ACC^*j*^—Yes), which enables further action, or the protocol is aborted. If both tests are passed, then output bits together with further bits from the SV-source (denoted by *t*) are introduced into an extractor to obtain final bits *S*.

Protocol I is the more basic one and employs just four devices, however, it needs an extractor that so far is only known to exist *implicitly* in the full range 0<*ɛ*<1/2 (alternatively, there is an explicit extractor that can be employed in the protocol, but then it can produce just one bit of randomness). This is overcome in our Protocol II, which is an extended version of Protocol I. It requires eight devices, but works with a fully explicit extractor for the whole range of *ɛ*, that is, even for arbitrarily weak sources. Protocol I is depicted in Fig. 1, whereas Protocol II is illustrated in Fig. 2. More precisely, our results can be formulated as the following two (informal) theorems, whose formal versions are given in the Supplementary Notes 5 and 6 (see Supplementary Theorems 20 and 27 for details).

*Theorem 1* [informal]: For every 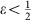, there is a protocol using an *ɛ*-SV source and four no-signalling devices with the following properties:

- Using the devices (*n*, log(1/*δ*)) times, the protocol either aborts or produces *n* bits, which are *δ*-close to uniform and independent of any side information (for example, held by an adversary).
- Local measurements on many copies of a four-partite entangled state, with (1−2*ɛ*) error rate, give rise to devices that do not abort the protocol with probability larger than 1−2^−Ω(*n*)^.

The protocol is non-explicit and runs in (*n*, log(1/*δ*)) time. Alternatively, it can use an explicit extractor to produce a single bit of randomness that is *δ*-close to uniform in (log(1/*δ*)) time.

*Theorem* 2 [informal]: For every 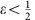, there is a protocol using an *ɛ*-SV source and eight no-signalling devices with the following properties:

- Using the devices 2^poly^ ^(*n*, log(1/*δ*))^ times, the protocol either aborts or produces *n* bits, which are *δ*-close to uniform and independent of any side information (for example, held by an adversary).
- Local measurements on many copies of a four-partite entangled state, with (1−2*ɛ*) error rate, give rise to devices that do not abort the protocol with probability larger than 1−2^−Ω(*n*)^.

The protocol is fully explicit and runs in 2^poly^^(*n*, lo*g*(1/*δ*))^ time.

### General setup

Let us first outline our general setup. We divide the bits from the SV-source into two parts. One part of the weakly random bits is fed into the devices, either as inputs or to choose some particular runs. Then, a test is performed on the inputs and outputs of the device. If the test is passed (denoted by an ‘ACC'), then the outputs and the other part of bits from the SV-source are fed into a randomness extractor—a deterministic function, whose output constitutes the almost ideal randomness. If the test is failed, the protocol is aborted.

The devices work as follows. A source produces four particle entangled states (of photons, atoms and so on). Each device performs a measurement on one of the four particles produced in each run. One of two measurements can be performed, which is decided by a binary input to each device. When the input is zero, one type of measurement is performed, whereas the other type is performed when the input is one. The measurements have binary outputs.

### Description of the protocols

Protocol I works as follows. A portion of bits from the SV-source is used as an input to the devices. A test is then performed, which amounts to checking whether a particular Bell inequality is violated to a certain specified level. The test consists of computing the following quantity


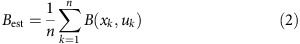


from *n* 4-tuples of inputs and outputs obtained in *n* runs (every *x*_*k*_ and *u*_*k*_ is of the form (***x***^1^,…***x***^4^) and (***u***^1^,…***u***^4^), respectively), and the explicit expression for *B*(*x*_*k*_, *u*_*k*_) is given in equation (7) of the Supplementary Note 2. The test is passed if *B*_est_≤*δ*. The parameter *δ*⩾0 can be interpreted as the noise level, that is, in the absence of noise we would observe *B*_est_=0. For the explicit form of the quantum state and measurements that achieve *B*_est_=0, see equations (8) and (9) of the Supplementary Note 2. If the test is failed, the protocol is aborted. If it is passed, the outputs of the devices and a second part of bits from SV-source are fed into an extractor, designed to extract randomness from two independent sources.

For Protocol II, we add a second group of four devices, operating in the same way as the original group. The runs (uses) of the devices from this second group are divided into blocks and a portion of bits from the SV-source is used to choose a block. The same test as in Protocol I is performed twice: first on all runs from the first group of devices, and then on the chosen block of runs from the second group. If the tests are passed, an extractor designed to extract randomness from three independent weak sources is applied to the three groups of variables: one from the SV-source, another formed by outputs from the first four devices and a third formed by the outputs from the chosen block of runs from the second group of four devices.

The merit of Protocol II is that it offers amplification of arbitrarily weak sources under a constant noise rate and with just a few devices. The probability of failure (failure occurs if the test was passed, but the output of the protocol is not random) scales as an inverse polynomial in the total number of runs. On the contrary, Protocol I has probability of failure exponentially small in the number of runs. Moreover, since for 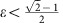 there exist explicit two source extractors^13^, Protocol I also gives exponential security for this range of *ɛ*. As a matter of fact, the field of extractors is being constantly developed. For instance, in a very recent development^14^ an extractor was found that, if used in our Protocol I, allows to draw one bit of randomness for an arbitrarily weak SV-source with exponential security.

Let us emphasize, that our protocols exhibit a strong security criterion called composable security^15^,^16^. This means that the obtained randomness can be securely used as an input to any other protocol. It also means that if an adversary Eve would in future learn part of the random bits output by the protocols (for example, by some espionage), the remaining bits would still be completely secure.

Last but not least, the security of our protocols relies on quantum-mechanical predictions, but can be verified by a person that either does not know or does not trust the quantum mechanical theory. Indeed, the security of our protocols is based on the very statistics of the outcomes of the device and the quantum mechanics is needed only to produce the required statistics. Moreover, Protocol I offers exponential security within such a paradigm.

In the proof sketched in the Methods section, we combine results from the classical theory of extractors obtained in refs 14, 17, 18, the recently discovered information-theoretic approach to the de Finetti theorem^19^ and the Azuma–Hoeffding inequality^20^.

### Additional remarks on assumptions

In the present work, we have also uncovered all the assumptions that were not necessarily explicit in the literature so far. Clearly, the minimal assumption one has to make is that of shielding devices, which means that the devices do not send signals to each other or to the external world and vice-versa. Indeed, if an eavesdropper can monitor our device instantaneously or if they can force it to behave in various ways, then any output of the device will be insecure. In addition, the device might have a transmitter inside that reports everything to the adversary. The shielding assumption is thus mandatory. Apart from this minimal assumption, we also make the constant device assumption, which has been either implicitly or explicitly used in almost all previous papers in this subject. This imposes that the device's behaviour is not correlated with the source of weak randomness (see Supplementary Note 3 for details). The only work that does not use this assumption is ref. 9, which, however, requires the use of many devices, thus falling into the above-described unpleasant alternative. Moreover, it also assumes that the adversary is restricted by the laws of quantum theory rather than just the shielding assumption.

Let us emphasize that in the classical world, the above assumptions are not enough to amplify randomness, as classical correlations are not strong enough to allow the verification procedure outlined above. Thus, the functioning of our protocols hinges on the intrinsic indeterminacy in the quantum description of nature.

## Discussion

We have presented realistic protocols for obtaining secure random bits from an arbitrarily weak Santha–Vazirani source. Both protocols use a finite number of devices, work even with correlations attainable by noisy quantum-mechanical resources and are composably secure against general no-signalling adversaries. The Protocol I uses four devices, and under the requirement of explicit extractors, can be either applied for a restricted range of epsilon to produce a non-zero rate of output randomness in polynomial time or for the entire range of epsilon to produce one bit of output randomness in polynomial time. The Protocol II uses eight devices and works for the entire range of epsilon to produce a non-zero rate of output randomness using an explicit extractor, however, it has the drawback of an exponential runtime. Important open questions for future research concern the relaxation of the constant device assumption (of independence between source and devices), and the development of protocols that can amplify general min-entropy sources of randomness in a secure manner against no-signalling adversaries. More open questions are raised in Supplementary Note 7.

## Methods

### Min-entropy sources and extractors

Before we sketch the proof of our result, let us describe one of the important ingredients of the proof—the min-entropy sources and randomness extractors. For given probability distribution {*p*_*i*_}, its min-entropy is defined as 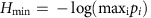, and a source which produces an *n*-bit distribution with min-entropy *k* is called an (*n*,*k*) min-entropy source. An example is the SV-source, whose min-entropy per bit is no smaller than 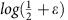. Although the randomness of a single min-entropy source cannot be amplified, it is known that one can classically amplify randomness from two or more independent min-entropy sources. The (deterministic) functions that do the job are called randomness extractors. We shall use the following results on extractors:

- There exists a (non-explicit) deterministic extractor that, given two independent sources of min-entropy larger than *h*, outputs *O*(*h*) bits 2^−*O*(*h*)^-close to uniform^17^.
- There exists an explicit extractor that, given three independent sources, one having min-entropy larger than *τn* (for any *τ*>0) and the other two larger than 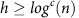 (with *c*>0 being an absolute constant), outputs *O*(*h*) bits 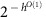 -close to uniform^21^. The extractor can be implemented in time (*n*,*h*).
- There exists an explicit extractor that, given two independent sources of min-entropy at least log^*C*^(*h*) for large enough constant *C* outputs 1 bit with error *h* ^−Ω(1)^ (ref. 14).

### Security of Protocol I: proof sketch

Let us now sketch the security proof of Protocol I (the full proof is provided in the Supplementary Note 5). The idea is the following: we prove that with high probability either, when conditioned on the inputs and upon acceptance (passing the test), the outputs of the devices form a min-entropy source or else the probability of acceptance is small. Thus, up to a small probability of failure (failure means that one accepts, but at the same time the devices do not constitute a min-entropy source), we have at our disposal two sources of weak randomness: the SV-source and the min-entropy source from the outputs of the devices. Because of the constant device assumption, when conditioned on the inputs, the two sources are independent, and we can apply a classical randomness extractor. Thus, the crux of the proof is to show that the outputs of devices constitute a min-entropy source. Here is where the Bell inequality comes into play.

Before going into more detail, let us introduce some notation. Consider *n* uses of each device, and let *x*, *u* be the outputs and inputs to the devices, respectively (the *u* come from the SV-source). Note that we have *x*=(*x*_1_,…,*x*_*n*_), *u*=(*u*_1_,…,*u*_*n*_), where every *x*_*k*_ and *u*_*k*_, for *k*∈{1,…,*n*}, is of the form ***x***=(***x***^1^,***x***^2^,***x***^3^,***x***^4^) and ***u***=(***u***^1^,***u***^2^,***u***^3^,***u***^4^), respectively. Let *w* and *z* be the input and output of the eavesdropper's device, respectively, and let *e* denote the side information possessed by the eavesdropper. Finally, we denote further bits drawn from the SV-source (apart from *u*) as *t*.

For any given *e*, the eavesdropper chooses optimally her input, which finally gives rise to a probability distribution *q*(*x*,*u*,*z*,*e*,*t*) satisfying certain conditions, given by equations (80)–(83) in Supplementary Note 4. The conditions come from our basic assumptions: shielding (equation (80) in Supplementary Note 5), constant devices (equation (81) in Supplementary Note 5) and causality (time-ordered no-signalling equation (82) in Supplementary Note 5). Let us denote by ACC the event of acceptance. We are thus interested in the min-entropy of the distribution *q*(*x*|*u*, *z*, *e*, *t*, ACC). We will actually prove that, with probability greater than 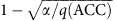, we have


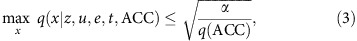


where *α*=2^−*Cn*^ with *C* being a constant, depending only on the SV-source parameter *ɛ* and the noise level *δ* (for details, see Proposition 19 in Supplementary Note 5). To this end, note that passing the test assures that the estimated value of the Bell quantity satisfies *B*_est_≤*δ*. Using the Azuma–Hoeffding theorem, we prove that, if the estimated Bell value is no greater than *δ*, then with probability 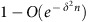, in a linear fraction of runs *μn*, with 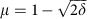, the ‘true' value of the SV-Bell quantity (conditioned on the history) is also small, that is, it is bounded by 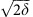 (see Lemma 9 in Supplementary Note 4 for details). The true value 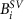 in the *i*-th run is here meant to be the average of *B*(***x***, ***u***) over the probability distribution in the run, conditioned on the history—the previous inputs and outputs, as well as Eve's variables *e*, *z*:


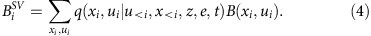


We then prove, using linear programming (see Lemma 10 and 11 in Supplementary Note 4 for details), that for any arbitrary distribution *p*(***x***, ***u***) of a single run, we have


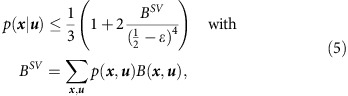


where ***u*** comes from the SV-source, so that 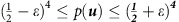. Applying this general relation to our case and using the Bayes rule, we obtain that for (*x*,*u*)∈ACC,


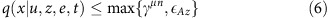


with 
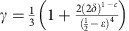
 for some constant *c*>0 (see Lemma 18 in Supplementary Note 5). Here 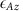 denotes the (small) failure probability of the estimation of the true SV-Bell value. For any given value of SV-source parameter *ɛ*, we see that there is a small enough value of the noise parameter *δ*, which ensures that *γ*<1, so that the probability *q*(*x*|*u*, *z*, *e*, *t*) in equation (6) is bounded by an exponentially (in *n*) small parameter, which in turn ensures that in the numerator of the right-hand-side of equation (3) there appears an exponentially decaying parameter *α*. The denominator therein appears due to the fact that we are conditioning on the event ACC in equation (3), unlike in equation (6). The technical details (some of them along the lines of ref. 20) that finally lead to equation (6) are presented in the proof of Theorem 20 in Supplementary Note 5 (ref. 22). This ends the sketch of the proof that our quantum device gives rise to a min-entropy source.

Now, as stated before, the constant device assumption assures that the min-entropy source obtained from the outputs of the device is independent of the bits from the SV-source that were not used as inputs. The application of a randomness extractor then gives the final output bits, although as noted earlier an explicit extractor is not known for all values of *ɛ*, except one that outputs a single bit of randomness^14^.

### Security of Protocol II: comment on the proof

In Protocol II, we manage to create three independent min-entropy sources, for which explicit extractors are known. To do this, we prove a version of the de Finetti theorem (see Lemma 13 in Supplementary Note 4 for details), which ensures that the outputs from the first 4-tuple of devices and the outputs belonging to the selected block of runs from the second 4-tuple are independent. As in Protocol I, each of the two sets of outputs constitutes a min-entropy source (conditioned on the two acceptances, as we perform separate tests for both sets). These, together with an unused portion of bits from the SV-source constitute three independent min-entropy sources, for which explicit extractors exist. The full proof of security of this protocol is provided in the Supplementary Note 6.

## Additional information

**How to cite this article**: Brandão, F. G. S. L. *et al*. Realistic noise-tolerant randomness amplification using finite number of devices. *Nat. Commun.* 7:11345 doi: 10.1038/ncomms11345 (2016).

## Supplementary Material

Supplementary Information Supplementary Figures 1-7, Supplementary Table 1, Supplementary Notes 1-7 and Supplementary References.
