# Supplementary material for: Realistic noise-tolerant randomness amplification using finite number of devices
Source: Nat Commun. 2016 Apr 21;7:11345. doi: 10.1038/ncomms11345 (PMC4844674; doi:10.1038/ncomms11345)
Supplement: Supplementary Information — Supplementary Figures 1-7, Supplementary Table 1, Supplementary Notes 1-7 and Supplementary References. [file ncomms11345-s1.pdf]

SUPPLEMENTARY FIGURES

Protocol I

1. The ε-SV source is used to choose the measurement settings $u_{\leq n}$ for the single device. The device produces output bits $x_{\leq n}$.
2. The parties perform an estimation of the violation of the Bell inequality in the device by computing the empirical average $L_n := \frac{1}{n}\sum_{i=1}^{n} B(x_i, u_i)$. The protocol is aborted unless $L_n \leq \delta$ (for $\delta > 0$ being a constant depending only on ε).
3. Conditioned on not aborting in the previous step, the parties apply the extractor from part (i) of Supplementary Lemma 3 to the sequence of outputs from the device and further n bits from the ε-SV source.

Supplementary Figure 1: **Protocol for device-independent randomness amplification from a single (four-partite) device with non-explicit extractor.** By $u_{\leq n}$ and $x_{\leq n}$ we mean $u_1, \ldots, u_n$ and $x_1, \ldots, x_n$, respectively. Function B in the definition of empirical average denotes an indicator vector for the Bell inequality. Extractor is here a deterministic function which maps bits from two independent sources of min-entropy into almost ideal random bits. The extractor in this protocol is non-explicit and produces a non-zero rate of output randomness in polynomial time (for the entire range of epsilon).

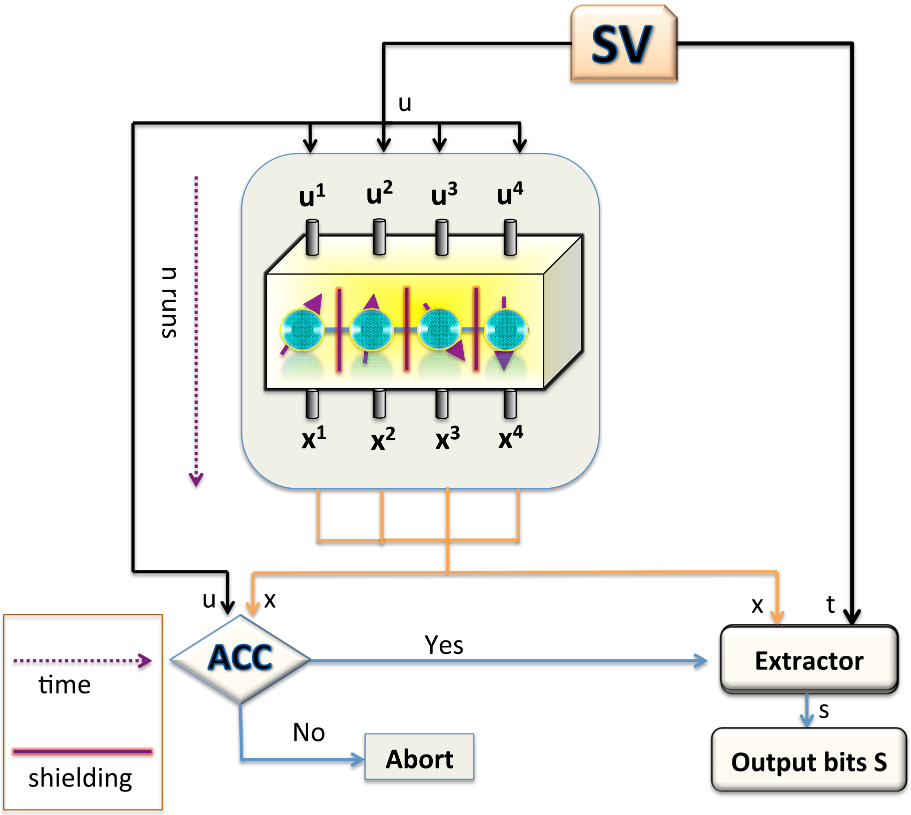

Supplementary Figure 2: **Illustration of the protocol for randomness amplification from 4 devices.** Devices are shielded one from another and used in a sequence of n runs. $u^1, \ldots u^4$ denote binary inputs in each run and they determine which measurement is made in a given run, while $x^1, \ldots, x^4$ are binary outputs of the measurements. If the test, preformed on the inputs and outputs of the device, is passed (denoted by ACC – Yes), then the outputs and another set of bits from the SV source (denoted by t) are introduced into an extractor in order to otain final output bits S. Black arrows mark the directions of bits from the SV source; orange ones indicate where output bits are introduced and blue arrows show possible alternatives: accepting the protocol and obtaining (supposedly random) bits or aborting the protocol.

Protocol II

1. The ε-SV source is used to choose the measurement settings $u_{\leq n}, v_{\leq M}$ for the 2 devices. The devices produce output bits $x_{\leq n}, y_{\leq M}$.
2. The measurements in device 2 are partitioned into N blocks of boxes each containing n boxes (so that $M = nN$).
3. The parties choose at random one block of boxes of size n from the second device (denoted by j), using bits from the ε-SV source.
4. The parties perform an estimation of the violation of the Bell inequality in the first device and in the chosen block of the second device by computing the empirical averages $L_n := \frac{1}{n}\sum_{i=1}^{n} B(x_i, u_i)$, $L_n := \frac{1}{n}\sum_{i=1}^{n} B(y_i^j, v_i^j)$. The protocol is aborted unless both of them are smaller or equal δ.
5. Conditioned on not aborting in the previous step, the parties apply the extractor from part (ii) of Supplementary Lemma 3 to the sequence of outputs from the first device and from the chosen block in the second device.

Supplementary Figure 3: **Protocol for device-independent randomness amplification from two (four-partite) devices with an explicit, and efficient, extractor.** By $u_{\leq n}$ and $x_{\leq n}$ we mean $u_1, \ldots, u_n$ and $x_1, \ldots, x_n$, respectively, and similarly for $v_{\leq M}$, $y_{\leq M}$. Function B in the definition of empirical average denotes an indicator vector for the Bell inequality. Extractor is here a deterministic function which maps bits from three independent sources of min-entropy into almost ideal random bits. It is explicit and produces a non-zero rate of output randomness for the entire range of epsilon, but it has the drawback of an exponential runtime.

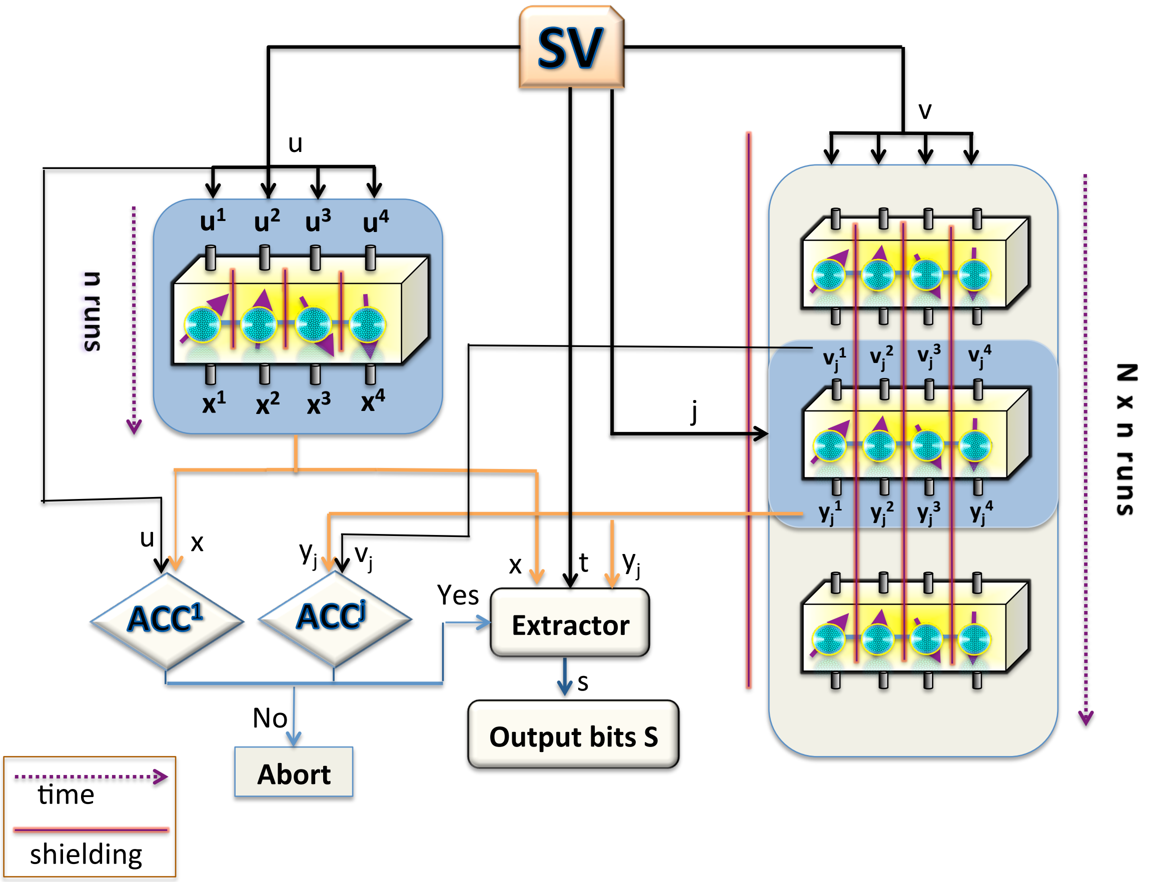

Supplementary Figure 4: **Illustration of the protocol for randomness amplification from 8 devices.** The 8 devices are shielded from each other and 1 block of n runs is performed sequentially on the first 4 devices, while and N blocks of n runs are performed sequentially on the second 4 devices. One of these N blocks, marked in blue, is selected by some portion of bits (denoted by j) from the SV source. $u^1, \ldots u^4, v_j^1, \ldots, v_j^4$ denote binary inputs in each run and they determine which measurement is made in the given run, while $x^1, \ldots, x^4, y_j^1, \ldots, y_j^4$ are binary outputs of the measurements. Black arrows indicate where the portions of bits form the SV source (u, v, j, t) are directed, while orange ones mark where output bits are introduced. The test is performed separately on inputs and outputs of devices of the first 4 devices and the chosen block from the second 4 devices. Blue arrows show possible alternatives: either the tests are passed (ACC^1 – Yes, ACC^j – Yes), which enables further action, or the protocol is aborted. If both tests are passed, then output bits together with further bits from the SV source (denoted by t) are introduced into an extractor to obtain final bits S.

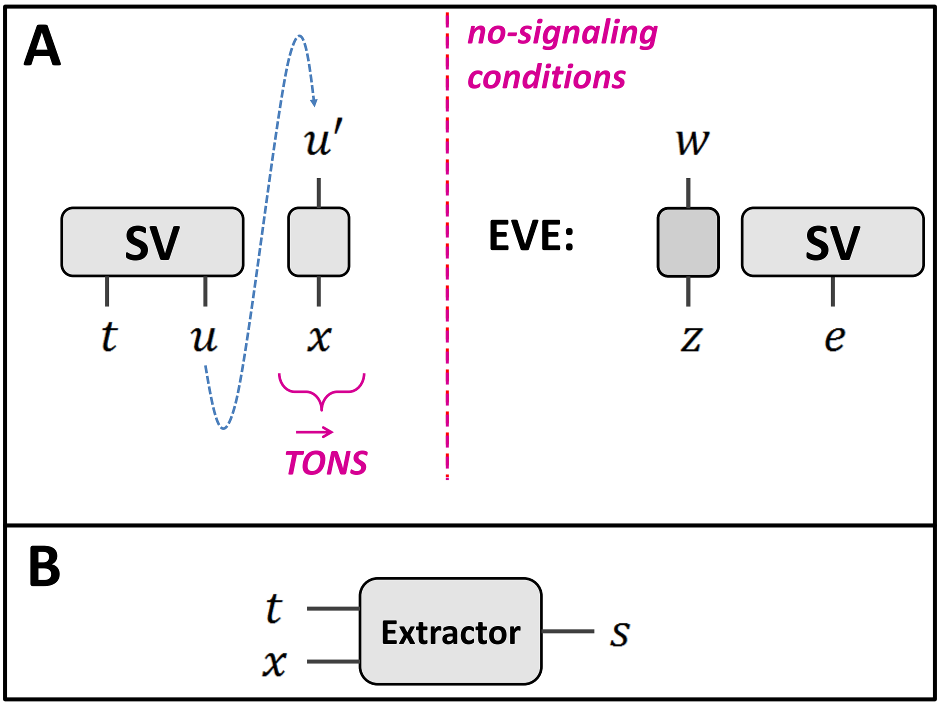

Supplementary Figure 5: **The setup for Protocol I.**
A. There are two portions of bits from the SV source: u and t. Honest parties and Eve share a no-signaling box $P(x,z|u',w)$ (no-signaling correlations are highlighted in the figure in pink), where (x,u') are the outputs and inputs of all the honest parties for the n runs of the protocol and (z,w) is the output-input pair of the adversary Eve. In addition, the box is time-ordered no-signaling (TONS). Honest parties obtain bits u from the SV source and use them as inputs to their box, so u' is set to be equal to u (which is illustrated by blue arrow). Eve has classical information e which are bits correlated to u, t.
B. Further bits t from the SV source, together with output bits x, are fed into the randomness extractor, obtaining the final output of the protocol $s = s(x,t)$.

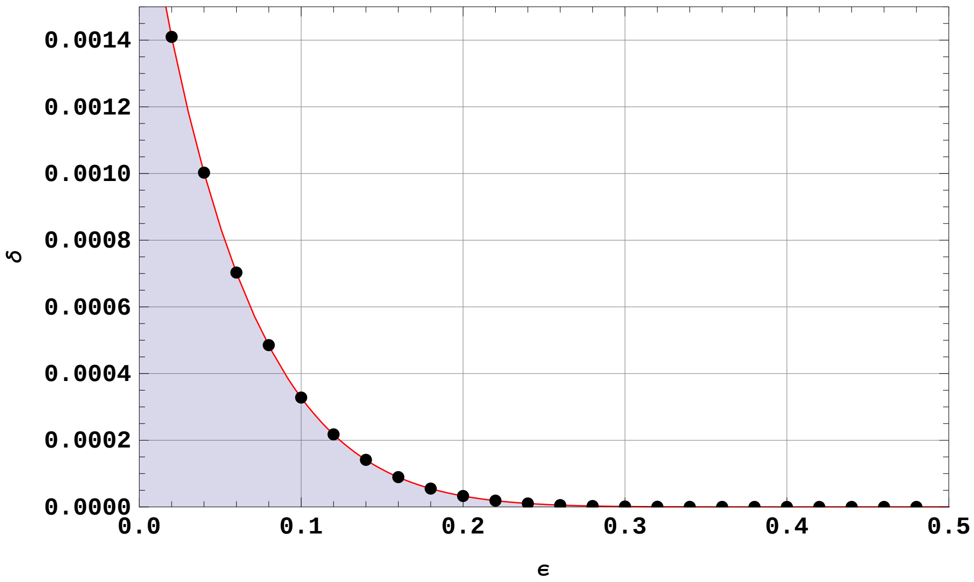

Supplementary Figure 6: **Trade-off between parameter ϵ of SV source and the amount of tolerated noise δ.** The analytical form of the tradeoff is given by the relation $\delta < \frac{1}{2}\left(\frac{1}{2} - \varepsilon\right)^8$ as shown in the text.

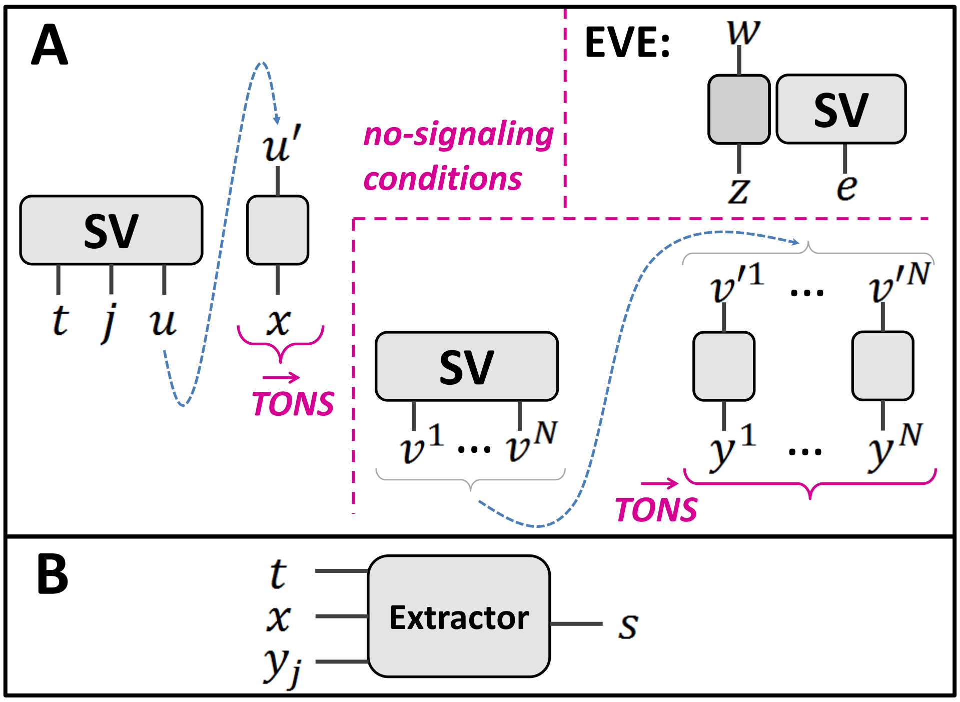

Supplementary Figure 7: **The setup for Protocol I.**
A. There are 4 portions of bits from the SV source: u, v, j, t, where $v = (v^1, \ldots, v^N)$. Honest parties have two no-signaling devices, whose components obey a time-ordered no-signaling (TONS) condition. They share with the adversary Eve a no-signaling box $P(x, y^1, \ldots, y^N, z|u', v'^1, \ldots, v'^N, w)$. No-signaling correlations are marked by pink dashed lines in the picture. Here we consider n uses of the first device with u' denoting the inputs for these, as well as N blocks of the second device (each with n uses) with $v'^1, \ldots, v'^N$ denoting the inputs for this device. Honest parties draw $u, v^1, \ldots, v^N$ from the SV source and use them as inputs to the devices (hence $u', v'^1, \ldots, v'^N$ are set equal to $u, v^1, \ldots, v^N$, as indicated by blue arrows). Variables $y^{j+1}, \ldots, y^N$ are then defined to be zero. The adversary Eve holds the classical information e about the bits drawn from the SV source.
B. The final output s of the protocol the honest parties compute as a function of x, y^j and t: $s = s(x, y^j, t)$.

SUPPLEMENTARY TABLES

	Source-Device Correlations	# Devices	Robustness	Eve	Comp.	Source	Public Source	Run time
Colbeck-Renner [1]	indep.	2	$\frac{1}{\mathrm{poly}(m)}$	NS	no	SV $\varepsilon < .08$	yes	$\mathrm{poly}(\frac{1}{\delta}, m)$
Gallego *et al* [2]	indep.	$\mathrm{poly}(\frac{1}{\delta}, m)$	$\frac{1}{\mathrm{poly}(m)}$	NS	yes	SV	yes	$2^{\mathrm{poly}(\log \frac{1}{\delta}, m)}$
**This Paper** Protocol I	indep.	4	$\Omega(1-2\varepsilon)$	NS	yes	SV	no	$\mathrm{poly}(\frac{1}{\delta}, m)$
**This Paper** Protocol II	indep.	8	$\Omega(1-2\varepsilon)$	NS	yes	SV	no	$2^{\mathrm{poly}(\log \frac{1}{\delta}, m)}$
Chung-Shi-Wu [3]	positive cond. min-entropy	$\mathrm{poly}(\frac{1}{\delta})$	$\Omega(1)$	Q	yes	$H_{\min}$	yes	$\mathrm{poly}(\log \frac{1}{\delta}, m)$
Mironowicz-Pawlowski * [4]	indep.	2	$\Omega(1)$	Q	no	SV	no	$\mathrm{poly}(\log \frac{1}{\delta}, m)$
Bouda *et al* * [5]	positive min-entropy	3	$\frac{1}{\mathrm{poly}(m)}$	Q	no	$H_{\min}$	yes	$\mathrm{poly}(\log \frac{1}{\delta}, m)$

Supplementary Table I: **Comparison of protocols for randomness amplification.**
In the table we used: m – number of output bits; δ – distance from uniform of output bits; NS and Q – standing for no-signaling and quantum adversaries, respectively. *The protocols proposed in [4] and [5] were not given a full security proof so far.

SUPPLEMENTARY NOTE 1: Outline of the Results and Comparison with Similar Work in the Area

We model a source of randomness as an ε-Santha-Vazirani (ε-SV) source, given by a probability distribution $p(\varphi_0, \ldots, \varphi_n)$ over bit strings such that

$$\begin{aligned} &0.5 - \varepsilon \leq P(\varphi_0) \leq 0.5 + \varepsilon, \\ &0.5 - \varepsilon \leq P(\varphi_i|\varphi_0, \ldots, \varphi_{i-1}) \leq 0.5 + \varepsilon \quad \text{for } 1 \leq i \leq n, \end{aligned} \tag{1}$$

where $\varepsilon \in [0, 0.5]$. The above equation is the only assumption on the source, which otherwise can be arbitrary. We address the following question: given an ε-SV source, is it possible to process the bits so that the quality of the randomness is improved? We propose two protocols achieving this task, as presented in the subsequent sections. In what follows, we first review the previous works on this subject.

Features of the Protocols and Comparison with Previous Works

In this section we compare our result with similar work in the area. The main protocols for randomness amplification proposed so far are summarized in Supplementary Table I. There are several aspects of a protocol for randomness amplification to be considered:

- **Source-Device Correlations:** In this paper (as in [1, 2]) we assume that the device shared by the honest parties and Eve is independent of the imperfect randomness source. A less demanding requirement is to require only that the source has randomness conditioned on the devices (this is the assumption considered in the work of Chung, Shi, and Wu [3] discussed below).
- **Number of Devices:** This is the total number of different no-signaling devices that are used in the protocol. For a protocol to be practical, it must involve only a small constant number of devices.
- **Robustness:** This is the amount of error per basic element that the protocol can withstand while still working correctly. A basic element is either a two-qubit gate, a qubit measurement, or the storage of one qubit for one time step. For the protocol to be practical it must tolerate a constant amount of noise per basic element.
- **Eavesdropper:** This represents the type of adversary under which the protocol is secure. It can be either a quantum adversary or a more powerful no-signaling adversary.
- **Composability:** This represents whether the protocol is composable (being secure even if Eve measures her part of the device after learning some of the output random bits).
- **Source:** Two types of sources have been considered so far: Santha-Vazirani (SV) sources and the more general min-entropy sources (where one only requires that the source has a certain amount of min-entropy).
- **Public Source:** This item makes a distinction between the way the bits of the random source are distributed. In a public source the bits are drawn from the source and then communicated to all the parties (including Eve). In a private source, the honest parties have exclusive access to a part of the source, and the adversary can only learn about the bits in this part from their correlations with the other parts of the source under the adversary's control.

- **Run Time:** This item quantifies the computational complexity of the protocol.

In their seminal work [1], Colbeck and Renner showed that non-local quantum correlations can be used to amplify the randomness of Santha-Vazirani (SV) sources that are sufficiently close to fully random. This result was later improved by Gallego *et al* [2], who gave a protocol using quantum non-local correlations to amplify general SV sources, as long as they are not deterministic. However, neither of the two protocols tolerate noise.

Another interesting protocol was proposed by Chung, Shi, and Wu [3] (after the first version of this paper appeared). While their protocol is sound only against quantum adversaries, it can amplify the randomness of any min-entropy source. Moreover, it has the distinguishing feature that the correct functioning of the protocol is guaranteed based only on the source having positive min-entropy conditioned on the (quantum) state of the devices. A drawback of the protocol is that it requires an unbounded number of devices.

Another interesting development was the work of Coudron and Yuen [6], who showed that any composable protocol can be made to have infinite rate (at the cost of increasing the number of devices by two and decreasing the robustness). Combining their result with ours, one obtains a protocol whose rate is infinite (the number of bits from the SV source only determines the output error).

Remarks to the results

Theorem 1 (Main Paper). An important assumption in Theorem 1 (Main Paper) is that the SV source is independent of the joint device shared by the honest parties and the adversary (see Supplementary Note 3 for details). The theorem is based on Protocol I, given in Supplementary Figure 1, using the four-partite Bell inequality given in Supplementary Note 2. See Theorem 18 in Supplementary Note 5 for a more precise formulation of Theorem 1 from Main Paper.

Theorem 1 has the drawback that the extractor used in Protocol I is non-explicit (we only know that it exists by the probabilistic method). Alternatively, there is an explicit extractor that can be employed in Protocol I that has been found recently [7], but then it can produce just one bit of randomness. We can improve on this aspect if we are willing to increase the number of no-signaling devices and worsen the efficiency of the protocol with the output error.

Theorem 2 (Main Paper). The protocol used in Theorem 2 (Main Paper) is given in Supplementary Figure 3. Its proof of correctness is analogous to the proof of Theorem 1, with a new ingredient that we show how to simulate two independent sources selecting subsystems at random (applying an analogue of the de Finetti Theorem of [8] to subsystems selected by the SV source). The advantage of this new step is that we have three independent sources and so can use known explicit and computationally efficient extractors.

Detailed description of protocols

Protocol I is simpler than the previous protocols [1, 2, 9]. It is given precisely in Supplementary Figure 1 and illustrated in Supplementary Figure 2, its rough structure is the following: first, one uses several bits from the SV source in order to choose inputs for four no-signaling boxes (each of which is reused many times). Then, one collects the outputs of boxes and, using the empirical data on inputs and outputs, decides whether to abort or not. Then, if the protocol is not aborted, one applies a randomness extractor (see Supplementary Note 2) to all output bits of the four devices

and to a further set of bits taken from the SV source. The output bits of the protocol, which by Theorem 1 (Main Paper) are close to fully random, are just the output bits of the extractor.

The proof of correctness of the protocol consists of three main steps.

(i) We show that by increasing the output error of the protocol (under the assumption that the source is private) it suffices to prove the security of the protocol considering an adversary that only has classical side-information about the devices of the honest parties. This is achieved in Supplementary Note 3.

(ii) We show that, conditioned on passing a certain test on the input and output of the four no-signaling boxes, with high probability over the given input, the output is a source of linear min-entropy. This is achieved in Proposition 17 in Supplementary Note 5.

(iii) We use known results on extractors for two independent sources.

Step (ii) is established by a sequence of implications (which have a similar flavor to the estimation of [10–12] for the related task of randomness expansion) as follows:

- In Supplementary Note 4 (describing the tools for the proof), we present an estimation procedure which ensures that with high probability the value of the Bell expression with settings chosen from the SV source is small (the Bell inequality we consider is such that the maximum possible violation corresponds to the zero value) for a linear fraction of boxes conditioned on previous inputs and outputs. This will follow from a simple application of Azuma's inequality.

- We then show that the small Bell value with settings chosen from the SV source implies that, for any setting, the probability of any output is bounded away from one. This is achieved by solving a linear program, analogously to the approach of [2, 9].

- Finally, we show that if a constant fraction of conditional boxes has probability of outputs bounded away from one, then, given the input, the output has linear min-entropy.

Step (iii) is an application of known results regarding extracting randomness from two independent min-entropy sources (see Supplementary Note 2). In our case the independence follows from our main assumption that the device is independent of the SV source.

It is instructive to point out where the quantum nature of the devices used in the protocol plays a role. It is in the second step that the non-local nature of quantum correlations is exploited. There, looking at the inputs and outputs of the devices, one can verify in a device-independent manner that the outputs must have been somewhat random. This is impossible classically without making further assumptions.

Protocol II. Protocol II is described in Supplementary Figure 3 and illustrated in Supplementary Figure 4. In the scenario where we require an explicit extractor a further set of four no-signaling boxes is taken into account. As before, bits from the SV source are input into these boxes and a test is performed on the empirical data of inputs and outputs. In this case, the randomness extractor is applied to the output bits of all eight no-signaling devices and a further set of bits from the SV source.

The proof of security of Protocol II is contained in Supplementary Note 6, with the main result stated in Supplementary Theorem 25. We need here an extra step to reduce the problem to having three independent sources of week randomness. Namely we show that we can effectively work with two independent devices (of four no-signaling parts each); where by independent we mean devices that, given any fixed inputs, produce uncorrelated outputs.

The idea is to adapt recent de Finetti theorems for no-signaling devices [8, 13] (based on information-theoretical methods) to the situation in which subsystems are selected from the SV source, instead of being selected uniformly at random. In Supplementary Lemma 11 we show that given two no-signaling devices, if we select at random (using the SV source) a block of uses from the second device among a sufficiently large number of blocks, this block of uses will be approximately uncorrelated with the first device.

SUPPLEMENTARY NOTE 2: Randomness extractors and Bell inequalities used in the paper

Randomness Extractors

Recall the definition of a min-entropy source:

Definition 1 (The min-entropy source). *The min-entropy of a random variable S is given by*

$$H_{\min}(S) = \min_{s \,\in\, supp(S)} \log \frac{1}{P(S=s)}. \tag{2}$$

When $S \in \{0,1\}^n$, it is called an $(n, H_{\min}(S))$.

From multiple weak sources of randomness, one can use independent source extractors [14] to extract nearly uniform random bits.

Definition 2 (Independent-source extractor). *An independent source extractor is a function $Ext : (\{0,1\}^n)^k \to \{0,1\}^m$ that acting on k independent $(n, H_{\min}(S))$ sources, outputs m bits which are ξ-close to uniform, i.e. for k independent $(n, H_{\min}(S))$ sources $S_1, \ldots, S_k$ we have*

$$\|Ext(S_1, \ldots, S_k) - U_m\|_1 \leq \xi, \tag{3}$$

where $\|.\|_1$ is the variational distance between the two distributions and U_m denotes the uniform distribution on m bits.

The results about extractors that we use are summarized in the following lemma.

Lemma 3 (Extractors Constructions).

(i) [15] There exists a (non-explicit) deterministic extractor that, given two independent sources of min-entropy larger than h, outputs $\Omega(h)$ bits $2^{-\Omega(h)}$-close to uniform.

(ii) [16] There exists an explicit extractor that given three independent sources, one having min-entropy larger than τn (for any $\tau > 0$) and the other – larger than $h \geq \log^c(n)$ (with $c > 0$ being a universal constant), outputs $\Omega(h)$ bits $2^{-h^{\Omega(1)}}$-close to uniform.The extractor can be implemented in time $\mathrm{poly}(n, h)$.

(iii) [7] There exists an explicit extractor that, given two independent sources of min-entropy at least $\log^C(h)$, for large enough constant C, outputs 1 bit with error $h^{-\Omega(1)}$.

When we say that the first extractor is non-explicit we mean that its existence is only guaranteed by the probabilistic method. Theorem 1 (Main Paper) uses the non-explicit extractor for two sources, stated in part (i), or alternatively to obtain just a single bit of randomness one can use the explicit extractor for two sources from part (iii), while Theorem 2 (Main Paper) uses Rao's extractor [16], stated in part (ii).

The Bell inequality

The inequality we consider involves four spatially separated parties with measurement settings $\mathbf{u} = \{\mathbf{u}^1, \mathbf{u}^2, \mathbf{u}^3, \mathbf{u}^4\}$ and respective outcomes $\mathbf{x} = \{\mathbf{x}^1, \mathbf{x}^2, \mathbf{x}^3, \mathbf{x}^4\}$. Each party chooses one of two measurement settings with two outcomes each, so that $\mathbf{u}^i \in \{0,1\}$ and $\mathbf{x}^i \in \{0,1\}$ for $i \in \{1,..,4\}$. The measurement settings for which non-trivial constraints are imposed by the inequality can be divided into two sets

$$U_0 = \{\{0001\},\{0010\},\{0100\},\{1000\}\} \quad \text{and} \quad U_1 = \{\{0111\},\{1011\},\{1101\},\{1110\}\}. \tag{4}$$

The inequality is then [17]

$$\sum_{\mathbf{x},\mathbf{u}} (\mathtt{I}_{\oplus_{i=1}^4 \mathbf{x}^i = 0}\, \mathtt{I}_{\mathbf{u}\in U_0} + \mathtt{I}_{\oplus_{i=1}^4 \mathbf{x}^i = 1}\, \mathtt{I}_{\mathbf{u}\in U_1})\, P(\mathbf{x}|\mathbf{u}) \geq 2, \tag{5}$$

where the indicator function $\mathtt{I}_L = 1$ if L is true and 0 otherwise. The local hidden variable bound is 2 and there exist no-signaling distributions that reach the algebraic limit of 0. For any no-signaling box represented by a vector of probabilities $\{P(\mathbf{x}|\mathbf{u})\}$, the Bell inequality may be written as

$$\mathbf{B}.\{P(\mathbf{x}|\mathbf{u})\} = \sum_{\mathbf{x},\mathbf{u}} B(\mathbf{x},\mathbf{u}) P(\mathbf{x}|\mathbf{u}) \geq 2, \tag{6}$$

where **B** is an indicator vector for the Bell inequality with $2^4 \times 2^4$ entries

$$B(\mathbf{x},\mathbf{u}) = \mathtt{I}_{\oplus_{i=1}^4 \mathbf{x}^i = 0}\, \mathtt{I}_{\mathbf{u}\in U_0} + \mathtt{I}_{\oplus_{i=1}^4 \mathbf{x}^i = 1}\, \mathtt{I}_{\mathbf{u}\in U_1}. \tag{7}$$

Consider the quantum state

$$|\Psi\rangle = \frac{1}{\sqrt{2}} \left(|\phi_-\rangle|\tilde{\phi}_+\rangle + |\psi_+\rangle|\tilde{\psi}_-\rangle \right), \tag{8}$$

where $|\phi_-\rangle = \frac{1}{\sqrt{2}}(|0\rangle|0\rangle - |1\rangle|1\rangle)$, $|\psi_+\rangle = \frac{1}{\sqrt{2}}(|0\rangle|1\rangle + |1\rangle|0\rangle)$, $|\tilde{\phi}_+\rangle = \frac{1}{\sqrt{2}}(|0\rangle|+\rangle + |1\rangle|-\rangle)$ and $|\tilde{\psi}_-\rangle = \frac{1}{\sqrt{2}}(|0\rangle|-\rangle - |1\rangle|+\rangle)$. Measurements in the X basis

$$\left\{ |+\rangle = \frac{1}{\sqrt{2}}(|0\rangle + |1\rangle),\ |-\rangle = \frac{1}{\sqrt{2}}(|0\rangle - |1\rangle) \right\} \tag{9}$$

correspond to $\mathbf{u}^i = 0$ and measurements in the Z basis

$$\{|0\rangle, |1\rangle\} \tag{10}$$

correspond to $\mathbf{u}^i = 1$ for each of the four parties $i \in \{1,\ldots,4\}$. These measurements on $|\Psi\rangle$ lead to the algebraic violation of the inequality, i.e., the sum of the probabilities appearing in the inequality is zero.

The reason for the choice of this Bell inequality is twofold. Firstly, as we have seen, there exist quantum correlations achieving the maximal no-signaling violation of the inequality, which implies that free randomness amplification starting from any initial ε of the SV source may be possible. Secondly, we will show (in Supplementary Lemma 9) that, for any measurement setting $\mathbf{u}$ out of the 2^4 possible settings in the inequality, the probability of any of the 2^4 output bit strings $\mathbf{x}$ is bounded away from one (for any no-signaling box) by a linear function of the uniform value of the Bell expression.

SUPPLEMENTARY NOTE 3: General set-up, assumptions on the box and composable security criterion

We have the following general setup. We consider the variable $\mathtt{sv}$ from the SV source and the box held by the honest parties and Eve. The devices held by the honest parties have input and output denoted by $\mathtt{in}$ and $\mathtt{out}$ and Eve's input and output are denoted $\mathtt{in}_E$ and $\mathtt{out}_E$, respectively.

The devices held by the honest parties are separated into m components with corresponding inputs and outputs $\mathtt{in}^i$ and $\mathtt{out}^i$ for $i \in [m]$; furthermore, for each device component we have l sequential runs with inputs and outputs denoted by $\mathtt{in}^i_k$ and $\mathtt{out}^i_k$ for $k \in [l]$, respectively. The variable $\mathtt{sv}$ is divided into three parts $(\mathtt{sv}_E, \mathtt{sv}_1, \mathtt{sv}_2)$, where $\mathtt{sv}_E$ is generated by Eve, while $\mathtt{sv}_1$ and $\mathtt{sv}_2$ are generated by the honest parties. The variables $\mathtt{sv}_1$ will be used as inputs to devices.

The honest parties will check whether $\mathtt{out}$ and $\mathtt{in}$ satisfy some joint acceptance condition (based on the violation of a particular Bell inequality); this acceptance event is denoted by ACC. Conditioned on acceptance, the variables $\mathtt{sv}_2$ together with the outputs $\mathtt{out}$ of devices will be hashed (using a multi-source extractor) to produce the output s - the output random bits. The variables $\mathtt{sv}_1$ and $\mathtt{sv}_2$ as well as the outputs $\mathtt{out}$ of the devices will be destroyed in the protocol (and in particular will remain hidden from Eve).

The initial state of the system will be defined as the following correlation box:

$$p(\mathtt{out}, \mathtt{out}_E, \mathtt{sv}_1, \mathtt{sv}_2, \mathtt{sv}_E | \mathtt{in}, \mathtt{in}_E) \tag{11}$$

We assume the following:

- **No-signaling assumptions:** The box satisfies the constraint of no-signaling between the honest parties and Eve

$$\begin{aligned} p(\mathtt{out}|\mathtt{in}, \mathtt{in}_E) &= p(\mathtt{out}|\mathtt{in}), \\ p(\mathtt{out}_E|\mathtt{in}, \mathtt{in}_E) &= p(\mathtt{out}_E|\mathtt{in}_E), \end{aligned} \tag{12}$$

  as well as a no-signaling condition between device components

$$p(\mathtt{out}^I|\mathtt{in}) = p(\mathtt{out}^I|\mathtt{in}^I) \quad \forall I \subseteq [m]. \tag{13}$$

  Each device component also obeys a time-ordered no-signaling ($\mathtt{tons}$) condition for the $k \in [l]$ runs performed on it:

$$p(\mathtt{out}^i_k|\mathtt{out}_E, \mathtt{in}^i, \mathtt{in}_E, \mathtt{sv}) = p(\mathtt{out}^i_k|\mathtt{out}_E, \mathtt{in}^i_{\le k}, \mathtt{in}_E, \mathtt{sv}) \quad \forall k \in [l] \tag{14}$$

  where $\mathtt{in}^i_{\le k} := \mathtt{in}^i_1, \ldots, \mathtt{in}^i_k$.

- **SV conditions:** The variables $(\mathtt{sv}_1, \mathtt{sv}_2, \mathtt{sv}_E)$ form an SV source, that is satisfy Eq. (1). In particular, $(\mathtt{sv}_2|\mathtt{sv}_1, \mathtt{sv}_E)$ is also an SV source.

- **Assumption A1:** The devices do not signal to the SV source, i.e. the distribution of $\mathtt{sv}$ is independent of the inputs $(\mathtt{in}, \mathtt{in}_E)$:

$$\sum_{\mathtt{out}, \mathtt{out}_E} p(\mathtt{out}, \mathtt{out}_E, \mathtt{sv}|\mathtt{in}, \mathtt{in}_E) = p(\mathtt{sv}) \quad \forall (\mathtt{sv}, \mathtt{in}, \mathtt{in}_E). \tag{15}$$

- **Assumption A2:** The box is fixed independently of the SV source:

$$p(\mathtt{out}, \mathtt{out}_E|\mathtt{in}, \mathtt{in}_E, \mathtt{sv}) = p(\mathtt{out}, \mathtt{out}_E|\mathtt{in}, \mathtt{in}_E) \quad \forall (\mathtt{out}, \mathtt{out}_E, \mathtt{sv}, \mathtt{in}, \mathtt{in}_E). \tag{16}$$

Assumption A1 is not very restrictive and is required to meaningfully describe the process of inputting a variable from the SV source into the box. Assumption A2, in turn, is more restrictive, although in our view is still a natural one. It is the quantum analogue of the problem of extracting randomness from one source and an independent (but unknown and arbitrary) channel. While it is easy to see that no randomness can be extracted in this classical setting, the situation is different considering non-local correlations. This assumption was also employed in the pioneering results [1, 2, 4], as well as in all other results on the topic apart from [3].

After $\mathtt{sv}_1$ is input as $\mathtt{in}$, we obtain the following box,

$$p(\mathtt{out},\mathtt{out}_E,\mathtt{sv}_1,\mathtt{sv}_2,\mathtt{sv}_E|\mathtt{in}_E) := p(\mathtt{out},\mathtt{out}_E,\mathtt{sv}_1,\mathtt{sv}_2,\mathtt{sv}_E|\mathtt{sv}_1,\mathtt{in}_E) \tag{17}$$

Due to assumption A1, which assures in particular no-signaling from $\mathtt{in}$ to $\mathtt{sv}_1$, this is a normalized probability distribution.

Conditioning on acceptance ACC and applying a hash function $s(\mathtt{out},\mathtt{sv}_2)$, one gets the following box

$$\begin{aligned} r(s,\mathtt{out}_E,\mathtt{sv}_E|\mathtt{in}_E) &:= p(s,\mathtt{out}_E,\mathtt{sv}_E|\mathtt{in}_E,\mathrm{ACC}) \\ &\equiv \sum_{\mathtt{sv}_1}\sum_{s(\mathtt{out},\mathtt{sv}_2)=s} p(\mathtt{out},\mathtt{out}_E,\mathtt{sv}_1,\mathtt{sv}_2,\mathtt{sv}_E,|\mathtt{in}_E,\mathrm{ACC}), \end{aligned} \tag{18}$$

where

$$p(\mathtt{out},\mathtt{out}_E,\mathtt{sv}_1,\mathtt{sv}_2,\mathtt{sv}_E|\mathtt{in}_E,\mathrm{ACC}) = \frac{p(\mathtt{out},\mathtt{out}_E,\mathtt{sv}_1,\mathtt{sv}_2,\mathtt{sv}_E|\mathtt{in}_E)}{\sum_{(\mathtt{out},\mathtt{sv}_1)\in\mathrm{ACC}} p(\mathtt{out},\mathtt{sv}_1|\mathtt{in}_E)} \tag{19}$$

The composable security criterion [18, 19] is defined in terms of the distance of $r(s,\mathtt{out}_E,\mathtt{sv}_E|\mathtt{in}_E)$ to an ideal box $r^{id} = \frac{1}{|S|}r(\mathtt{out}_E,\mathtt{sv}_E|\mathtt{in}_E)$, with $r(\mathtt{out}_E,\mathtt{sv}_E|\mathtt{in}_E) = \sum_s r(s,\mathtt{out}_E,\mathtt{sv}_E|\mathtt{in}_E)$. The distance is the standard variational distance between probability distributions maximized over all possible measurements applied to the box. Since the most general measurement that Eve can apply is to look at the register $\mathtt{sv}_E$, as well as the register s, and input $\mathtt{in}_E$ that may depend on both of them, we have:

$$d_c = \sum_{s,\mathtt{sv}_E}\max_{\mathtt{in}_E}\sum_{\mathtt{out}_E}\left|r(s,\mathtt{out}_E,\mathtt{sv}_E|\mathtt{in}_E) - \frac{1}{|S|}r(\mathtt{out}_E,\mathtt{sv}_E|\mathtt{in}_E)\right|. \tag{20}$$

One can rewrite it as follows

$$d_c = \sum_{s,\mathtt{sv}_E}\max_{\mathtt{in}_E}\sum_{\mathtt{out}_E} r(\mathtt{out}_E,\mathtt{sv}_E|\mathtt{in}_E)\left|r(s|\mathtt{out}_E,\mathtt{sv}_E,\mathtt{in}_E) - \frac{1}{|S|}\right| \tag{21}$$

Rewriting it in terms of the box p we obtain

$$d_c = \sum_{s,\mathtt{sv}_E}\max_{\mathtt{in}_E}\sum_{\mathtt{out}_E} p(\mathtt{out}_E,\mathtt{sv}_E|\mathtt{in}_E,\mathrm{ACC})\left|p(s|\mathtt{out}_E,\mathtt{sv}_E,\mathtt{in}_E,\mathrm{ACC}) - \frac{1}{|S|}\right|. \tag{22}$$

The composable secure definition says that the protocol is ϵ-secure if $d_c \leq \epsilon$, for a chosen error ϵ. It guarantees that even if part of s is given to Eve, the rest is still secure.

We now note that

$$d_c \leq |S|\sum_{\mathtt{sv}_E}\max_{\mathtt{in}_E}\sum_{\mathtt{out}_E} p(\mathtt{out}_E,\mathtt{sv}_E|\mathtt{in}_E,\mathrm{ACC})\sum_s\left|p(s|\mathtt{out}_E,\mathtt{sv}_E,\mathtt{in}_E,\mathrm{ACC}) - \frac{1}{|S|}\right|. \tag{23}$$

In our proofs we will use this estimate and therefore will have to handle the extra factor of the size of the output $|S|$ in the error. The benefit will be that one can fix the measurement of Eve beforehand. In particular this allows us to use an extractor sound only with respect to a classical adversary and nevertheless obtain security under a no-signaling adversary.

We note that apart from the fact that we use a two-source extractor rather than hashing a single source, the payment of $|S|$ and the assumption of private source enables to circumvent related results on the limitations of privacy amplification under time-ordered no-signaling constraints [20]. We further note that assuming Eve is quantum, in other problems such as quantum key distribution and randomness expansion, it is possible to avoid the increase of the output error by $|S|$ using extractors that are sound against quantum side-information. The fact that an extractor that outputs n bits with error ϵ sound against classical side information is also sound against quantum (or even no-signaling) side information with error $2^n\epsilon$ is well known (see e.g. [21]). However, in many applications this error blow up is prohibitive. It turns out that in our approach we can afford it by tracing out a large fraction of the number of output bits.

SUPPLEMENTARY NOTE 4: Detailed description of mathematical tools necessary for proofs of main theorems

In this section we give the tools we will employ proving the correctness of Protocols I and II.

Estimation of the Bell value

In this section we first show, that with high probability, the arithmetic average of mean values for conditional boxes is close to the observed value. As a corollary, we obtain that if the average is small, then with high probability for a linear fraction of all boxes the mean will be small too. It has a similar flavor to previous results [10–12] obtained in the context of the related problem of randomness expansion.

Remark 4. *In the proof of Protocol I the variables W_i will be interpreted as $W_i = (x_i, u_i)$, for $i \in \{1, \ldots, n\}$, where any x_i and u_i are of the form of $\boldsymbol{x} = (\boldsymbol{x}^1, \ldots, \boldsymbol{x}^4)$ and $\boldsymbol{u} = (\boldsymbol{u}^1, \ldots, \boldsymbol{u}^4)$, respectively, both introduced when defining the Bell inequality in Supplementary Note 2, and $W_0 = (z, e)$. When we consider the general set-up described in Supplementary Note 3, we just set $\mathtt{out} := x_i$, $\mathtt{in} := u_i$, $\mathtt{out}_E := z$ and $\mathtt{in}_E := w$ (see Supplementary Note 5 for details).*

As for Protocol II, the following lemma will be applied twice. For the first time, we will take $W_i = (x_i, u_i)$ for $i \in \{1, \ldots, n\}$ and $W_0 = (z, v^j, M_j, e)$. For the second time, we will take $W_i = (y_i^j, v_i^j)$ for $i \in \{1, \ldots, n\}, j \in \{1, \ldots, N\}$, and $W_0 = (z, u, M_j, e)$ (see Supplementary Note 6 for the definitions of all variables used above).

The function B_i will be the same for both those cases, for all $i \in \{1, \ldots, n\}$, given by Eq. (7).

Lemma 5. *Consider arbitrary random variables W_i, for $i = 0, 1, \ldots, n$, and binary random variables B_i, for $i = 1, \ldots n$, that are functions of W_i, i.e. $B_i = f_i(W_i)$ for some functions f_i. Let us denote $\overline{B}_i = \mathbb{E}(B_i | W_{i-1}, \ldots, W_1, W_0)$, for $i = 1, \ldots, n$ (i.e. $\overline{B}_i$ are conditional expectation values). Define, for $k = 1, \ldots, n$, the empirical average*

$$L_k = \frac{1}{k} \sum_{i=1}^{k} B_i \tag{24}$$

and the arithmetic average of conditional expectation values

$$\overline{L}_k = \frac{1}{k}\sum_{i=1}^{k}\overline{B}_i. \tag{25}$$

Then we have

$$P(|L_n - \overline{L}_n| \geq s) \leq 2e^{-n\frac{s^2}{2}}. \tag{26}$$

To prove Supplementary Lemma 5, we need to state the Azuma-Hoeffding inequality. Let $X_0, \ldots, X_k$ and $W_0, \ldots, W_k$ be two sequences of random variables. Then $X_0, \ldots, X_k$ is said to be a martingale with respect to $W_0, \ldots, W_k$ if, for all $0 \leq i \leq k$, $\mathbb{E}|X_i| < \infty$ and $\mathbb{E}(X_i|W_0, \ldots, W_{i-1}) = X_{i-1}$.

Lemma 6. *(Azuma-Hoeffding) Suppose $X_0, \ldots, X_k$ is a martingale with respect to $W_0, \ldots, W_k$, and that $|X_{l+1} - X_l| \leq c_l$ for all $0 \leq l \leq k-1$. Then, for all positive reals t,*

$$P\left(|X_k - X_0| \geq t\right) \leq 2\exp\left(-\frac{t^2}{2\sum_{l=1}^{k} c_l^2}\right). \tag{27}$$

Now we can prove Supplementary Lemma 5.

Proof. Define $X_0 = 0$, $X_l = l(L_l - \overline{L}_l)$. Let us show that $\{X_i\}_{i=0}^{n}$ and $\{W_i\}_{i=0}^{n}$ satisfy the assumptions of Supplementary Lemma 6. First,

$$|X_l - X_{l-1}| = |\overline{B}_l - B_l| \leq 1, \tag{28}$$

since B_l is binary and $0 \leq \overline{B}_l \leq 1$. Let us now check that $\{X_l\}_{l=0}^{n}$ is a martingale with respect to $\{W_l\}_{i=0}^{n-1}$. We have $\mathbb{E}|X_l| \leq l < \infty$. Moreover, for $l \geq 2$ we have

$$\mathbb{E}(X_l|W_{l-1}, \ldots, W_0) = \sum_{i=1}^{l}\mathbb{E}(B_i|W_{l-1}, \ldots, W_0) - \sum_{i=1}^{l}\mathbb{E}(\overline{B}_i|W_{l-1}, \ldots, W_0) \tag{29}$$

$$= \sum_{i=1}^{l}\mathbb{E}(B_i|W_{l-1}, \ldots, W_0) - \sum_{i=1}^{l}\overline{B}_i \tag{30}$$

$$= \sum_{i=1}^{l-1}\mathbb{E}(B_i|W_{l-1}, \ldots, W_0) - \sum_{i=1}^{l-1}\overline{B}_i \tag{31}$$

where we used the property

$$\mathbb{E}(\mathbb{E}(A|B)|BC) = \mathbb{E}(A|B) \tag{32}$$

for random variables A, B, C. Now let us note that since B_i is a function of W_i, we get for $i \leq l-1$

$$\mathbb{E}(B_i|W_{l-1}, \ldots, W_1, W_0) = B_i \tag{33}$$

Thus the last line of Eq. (31) is equal to X_{l-1}, so that we have

$$\mathbb{E}(X_l|W_{l-1}, \ldots, W_1, W_0) = X_{l-1} \tag{34}$$

for $l = 2, \ldots, n$. For $l = 1$ one may verify Eq.(34) by checking directly that $\mathbb{E}(X_1|W_0) = 0$, being hence equal to X_0 defined to be zero. Indeed, in the latter case $X_1 = B_1 - \overline{B}_1$ where $\overline{B}_1 = \mathbb{E}(B_1|W_0)$, hence using (32) we get $\mathbb{E}(B_1 - \overline{B}_1|W_0) = \mathbb{E}(B_1|W_0) - \mathbb{E}(\mathbb{E}(B_1|W_0)|W_0) = 0$. Now, we apply Supplementary Lemma 6 with $c_l = 1$, and obtain the inequality (26). □

We also note the following useful fact.

Lemma 7. *If the arithmetic average $\overline{L}_n$ of n conditional means in Eq.(25) satisfies $\overline{L}_n \leq \delta$ for some parameter $\delta > 0$, then in at least $(1-\sqrt{\delta})n$ of positions i we have $\overline{B}_i \leq \sqrt{\delta}$*

Proof. Assume that $\overline{L}_n = \frac{1}{n}\sum_{i=1}^{n} \overline{B}_i \leq \delta$ with $\overline{B}_i \geq 0 \ \ \forall i$. Consider the set $I := \{i | \overline{B}_i \geq \frac{\delta}{\gamma}\}$. Then, $\frac{|I|}{n}\frac{\delta}{\gamma} \leq \delta$ so that $|I| \leq n\gamma$. Choosing $\gamma = \sqrt{\delta}$ we obtain that the fraction μ of positions i with value $\overline{B}_i < \sqrt{\delta}$ is given by $\mu = (1 - \frac{|I|}{n}) \geq 1 - \sqrt{\delta}$. □

Randomness of individual box from good Bell value

In the previous subsection, we have seen that if the observed Bell value is small, then there is a linear number of conditional boxes, with small Bell value (with settings chosen from an SV source). In Supplementary Lemma 10, we will show that in order to obtain a min-entropy source we need to ensure that a constant fraction of conditional boxes has randomness. In this section we will tie up the two observations by arguing that the randomness of a box is ensured if the value of the Bell expression with inputs taken from an SV source is small.

Let U denote all the settings appearing in the Bell expression. We consider first the uniform Bell value

$$\overline{B}^{U} := \frac{1}{|U|}\mathbf{B}.\{P(\mathbf{x}|\mathbf{u})\} = \frac{1}{|U|}\sum_{\mathbf{x},\mathbf{u}} B(\mathbf{x},\mathbf{u})P(\mathbf{x}|\mathbf{u}), \tag{35}$$

where $|U|$ denotes the cardinality of U, i.e. the total number of settings in the Bell expression. If the Bell function $B(\mathbf{x},\mathbf{u})$ is properly chosen, one can prove using linear programming that if $\overline{B}^{U}$ is small, the probabilities of any outputs are bounded away from 1. However, since our inputs to each device are chosen using an SV source, we will be only able to estimate the value of the following expression

$$\overline{B}^{SV} = \sum_{\mathbf{x},\mathbf{u}} \nu(\mathbf{u})B(\mathbf{x},\mathbf{u})P(\mathbf{x}|\mathbf{u}), \tag{36}$$

where $\nu(\mathbf{u})$ is the distribution from an (unknown) SV source. We will show that for a suitably chosen Bell function, when the latter expression is small, the former is also small which implies randomness.

In the following lemma, we prove the relation between the SV Bell value and randomness for a particular Bell inequality given by Eq. (5). It says that for an SV source of arbitrary $\varepsilon \neq \frac{1}{2}$, if the SV Bell value is small enough, the probability of any outcome is bounded away from 1.

Lemma 8. *Consider a four-partite no-signaling box $P(\boldsymbol{x}|\boldsymbol{u})$ satisfying*

$$\overline{B}^{SV} \leq \delta, \tag{37}$$

where $\overline{B}^{SV}$ is given by Eq. (36) with $B(\boldsymbol{x},\boldsymbol{u})$ given by Eq. (7). Then, for any measurement setting $\boldsymbol{u}^*$ and any output $\boldsymbol{x}^*$, we have

$$P\left(\boldsymbol{x}^*|\boldsymbol{u}^*\right) \leq \frac{1}{3}\left(1 + \frac{2\delta}{(\frac{1}{2}-\epsilon)^4}\right). \tag{38}$$

Proof. From the definition of an ε-SV source we have

$$(\frac{1}{2}-\varepsilon)^4 \leq \nu(\mathbf{u}) \leq (\frac{1}{2}+\varepsilon)^4. \quad (39)$$

so that

$$\frac{1}{(\frac{1}{2}+\varepsilon)^4|U|}\overline{B}^{SV} \leq \overline{B}^{U} \leq \frac{1}{(\frac{1}{2}-\varepsilon)^4|U|}\overline{B}^{SV} \quad (40)$$

Then, the claim follows from Supplementary Lemma 9, relating $\overline{B}^{U}$ with $P\left(\mathbf{x}^*|\mathbf{u}^*\right)$ by use of linear programming. □

Bounding output probabilities by linear programming

Let us show that for the specific Bell inequality we consider, when the value of the Bell expression is small there is weak randomness. Consider a four-partite no-signaling box $P(\mathbf{x}|\mathbf{u})$ that obtains a value δ for the Bell expression in Eq. (5). The following lemma shows that for any measurement setting $\mathbf{u}$, the probability of any outcome $\mathbf{x}$ is bounded from above by a function of δ.

Lemma 9. *Consider a four-partite no-signaling box $P(\boldsymbol{x}|\boldsymbol{u})$ satisfying*

$$\overline{B}^{U} := \frac{1}{|U|}\boldsymbol{B}.\{P(\boldsymbol{x}|\boldsymbol{u})\} \leq \frac{\delta}{|U|}, \quad (41)$$

for some $\delta \geq 0$, with $\boldsymbol{B}$ the indicator vector for the Bell expression in Eq. (5) and $|U| = 16$ the number of settings in the Bell expression. For any measurement setting $\boldsymbol{u}^*$ and any output $\boldsymbol{x}^*$, we have

$$P\left(\boldsymbol{x}^*|\boldsymbol{u}^*\right) \leq \frac{1+2\delta}{3}. \quad (42)$$

Proof. Consider any measurement setting $\mathbf{u}^*$ and any corresponding output $\mathbf{x}^*$ for this setting. Then $P\left(\mathbf{x}^*|\mathbf{u}^*\right)$ can be computed by the following linear program

$$\begin{aligned} P\left(\mathbf{x}^*|\mathbf{u}^*\right) \;=\; & \max_{\{P\}} : M^T_{\mathbf{x}^*,\mathbf{u}^*}\{P(\mathbf{x}|\mathbf{u})\} \\ & s.t. \;\; A\{P(\mathbf{x}|\mathbf{u})\} \leq c. \end{aligned} \quad (43)$$

Here, the indicator vector $M_{\mathbf{x}^*,\mathbf{u}^*}$ is a $2^4 \times 2^4$ element vector with entries $M_{\mathbf{x}^*,\mathbf{u}^*}(\mathbf{x},\mathbf{u}) = \mathbb{I}_{\mathbf{x}=\mathbf{x}^*}\mathbb{I}_{\mathbf{u}=\mathbf{u}^*}$. The constraint on the box $\{P(\mathbf{x}|\mathbf{u})\}$ written as a vector with $2^4 \times 2^4$ entries is given by the matrix A and the vector c. These encode the no-signaling constraints between the four parties, the normalization and the positivity constraints on the probabilities $P(\mathbf{x}|\mathbf{u})$. In addition, A and c also encode the condition that $\mathbf{B}.\{P(\mathbf{x}|\mathbf{u})\} \leq \delta$ with δ the bound on the Bell value for the box. Analogous programs can be formulated for each of the 2^4 measurement settings appearing in the Bell inequality in Eq. (5) and each of the 2^4 corresponding outputs.

The solution to the primal linear program in Eq. (43) can be bounded by any feasible solution to the dual program which is written as

$$\begin{aligned} & \min_{\lambda_{\mathbf{x}^*,\mathbf{u}^*}} : c^T\lambda_{\mathbf{x}^*,\mathbf{u}^*} \\ & s.t. \;\; A^T\lambda_{\mathbf{x}^*,\mathbf{u}^*} = M_{\mathbf{x}^*,\mathbf{u}^*}, \\ & \qquad \lambda_{\mathbf{x}^*,\mathbf{u}^*} \geq 0. \end{aligned} \quad (44)$$

For each $\{\mathbf{u}^*, \mathbf{x}^*\}$, we find a feasible $\lambda_{\mathbf{x}^*,\mathbf{u}^*}$ satisfying the constraints to the dual program above that gives $c^T \lambda_{\mathbf{x}^*,\mathbf{u}^*} \leq \left(\frac{1+2\delta}{3}\right)$. We therefore obtain by the duality theorem of linear programming that

$$P\left(\mathbf{x}^* | \mathbf{u}^*\right) \leq \left(\frac{1+2\delta}{3}\right), \tag{45}$$

which is the required bound. □

A min-entropy source from randomness of conditional boxes

In this section we show that if a device is such that a linear number of conditional boxes have randomness (in the weak sense that the probability of their outputs is bounded away from one), then the distribution on outputs constitutes a min-entropy source. The considerations in this section will be applicable to any of the devices $j \in [k]$ and any chosen block. Therefore we will skip the indices for simplicity.

Let any sequence $(x_1, u_1, \dots, x_n, u_n)$ be such that x_i and u_i, $i \in \{1, \dots, n\}$ are of the form of $\mathbf{x} = (\mathbf{x}^1, \dots, \mathbf{x}^4)$ and $\mathbf{u} = (\mathbf{u}^1, \dots, \mathbf{u}^4)$, respectively, both introduced in the definition of the Bell inequality in Supplementary Note 2. We will show that if, with large probability over sequences $(x_1, u_1, \dots, x_n, u_n)$, a constant fraction of those boxes has, for any setting, probability of every output bounded away from 1, then the total probability distribution is close in variational distance to a min-entropy source (see [10–12] for a similar result in the context of randomness expansion).

We first prove that, if this happens for all sequences (i.e. with probability 1), then the total box is a min-entropy source itself and subsequently we consider the case when the probability is close to 1 (not exactly 1).

Lemma 10. *Fix any measure P on the space of sequences $(x_1, u_1, \dots, x_n, u_n)$. Suppose that for a sequence $(x_1, u_1, \dots, x_n, u_n)$, there exists $K \subseteq [n]$ of size larger than μn, such that for all $l \in K$ the conditional boxes $P_{x_{<l},u_{<l}}(x_l|u_l)$ satisfy*

$$P_{x_{<l},u_{<l}}(x_l|u_l) \leq \gamma, . \tag{46}$$

Then, $P(x_1, \dots, x_n|u_1, \dots, u_n)$ satisfies

$$P(x_1, \dots, x_n|u_1, \dots, u_n) \leq \gamma^{\mu n} \tag{47}$$

Proof. The proof proceeds by successive application of the Bayes rule and the time-ordered no-signaling structure, i.e.

$$\begin{aligned} P(x_1, \dots, x_n|u_1, \dots, u_n) &= P(x_1|u_1)P(x_2|u_2, x_1, u_1) \\ &\quad \dots P(x_n|u_n, x_{n-1}, u_{n-1}, \dots, x_1, u_1), \end{aligned} \tag{48}$$

where we have used the fact that the outputs of the l-th box can depend only upon the inputs and outputs of the previous boxes due to the time-ordered structure of the boxes (see Eq. (14)). Now, due to the assumption that at least μn of the conditional boxes $P_{x_{<l},u_{<l}}(x_l|u_l)$ satisfy Eq. (46), we have that

$$P(x_1, \dots, x_n|u_1, \dots, u_n) \leq \gamma^{\mu n}. \tag{49}$$

□

Imposing independence between devices by a de Finetti bound with limited randomness

Consider two devices, the first consisting of n boxes and the second consisting of N_2 blocks of n boxes each. In this section, we show that, for suitable choice of N_2, the boxes from the first device are close to being uncorrelated with the boxes in a block chosen from the second device using an ε-SV source. The lemma is based on the information-theoretic approach of [8, 13] for proving the de Finetti theorems for quantum states and no-signaling distributions.

We denote the box by $P(X^1, X^2_{\le N_2}|U^1, U^2_{\le N_2})$, where the superscript denotes the device and the subscript denotes the block of uses of the device. Capital letters denote the inputs and outputs for a set of n boxes so that $X^1 = (x^1_1, \ldots, x^1_n)$ and $X^2_{\le N_2} = (x^2_{1,1}, \ldots, x^2_{n,1}, \ldots, x^2_{1,N_2}, \ldots, x^2_{n,N_2})$ with the second subscript denoting the block. Note that any x^1_k or $x^2_{k,l}$ for $k \in \{1, \ldots, n\}$, $l \in \{1, \ldots, N_2\}$ are of the form $\mathbf{x} = (\mathbf{x}^1, \ldots, \mathbf{x}^4)$ introduced in Supplementary Note 2. Similarly, u^1_k and $u^2_{k,l}$, for $k \in \{1, \ldots, n\}$ and any $l \in \{1, \ldots, N_2\}$, are of the form $\mathbf{u} = (\mathbf{u}^1, \ldots, \mathbf{u}^4)$.

Lemma 11. *Let $P(X^1, X^2_{\le N_2}|U^1, U^2_{\le N_2})$ satisfy the no-signaling conditions, i.e. Eqs. (12)-(14), with output and input alphabets Σ and Λ, respectively (i.e. $P : \Sigma^{\times (N_2+1)n} \times \Lambda^{\times (N_2+1)n} \to \mathbb{R}^+$). The distribution P represents two devices with the first containing n boxes and the second N_2 blocks of n boxes each. Let $A_2 \in [N_2]$ and $(U^1, U^2_{\le N_2})$ be chosen from an ε-SV source; we write $\nu(j, U^1, U^2_{\le N_2})$ and the distribution ν satisfies condition (1). Then, we have*

$$\mathop{\mathbb{E}}_{(j,U^1,U^2_{\le N_2})\sim\nu} \; \mathop{\mathbb{E}}_{X^2_{<j}\sim P(.|U^1,U^2_{\le N_2})} \left\| \tilde{P}(X^1, X^2_j|U^1, U^2_j) - \tilde{P}(X^1|U^1) \otimes \tilde{P}(X^2_j|U^2_j) \right\| \le \sqrt{2\ln(2) N_2^{\log(1+2\varepsilon)} \frac{n \log|\Sigma|}{N_2}}, \tag{50}$$

where $\tilde{P}$ is the conditional box given the inputs $U^2_{<j}$ and outputs $\mathbf{X}^2_{<j}$ of all prior boxes to the ones in the j-th block, i.e.

$$\tilde{P}(X^1, X^2_j|U^1, U^2_j) := P_{X^2_{<j}, U^2_{<j}}(X^1, X^2_j|U^1, U^2_j). \tag{51}$$

Proof. Using the upper bound on mutual information $I(A : B) \le \min(\log|A|, \log|B|)$ and the chain rule $I(A : BC) = I(A : B) + I(A : C|B)$, we have that for every distribution ν

$$\begin{aligned}
n \log|\Sigma| &\ge \mathop{\mathbb{E}}_{U^1,U^2_{\le N_2}\sim\nu} I(X^1 : X^2_{\le N_2})_{P(\cdot|U^1,U^2_{\le N_2})} \\
&= \mathop{\mathbb{E}}_{U^1,U^2_{\le N_2}\sim\nu} \left(I(X^1 : X^2_1)_{P(\cdot|U^1,U^2_{\le N_2})} + \ldots + I(X^1 : X^2_{N_2}|X^2_{<N_2})_{P(\cdot|U^1,U^2_{\le N_2})} \right) \\
&= \mathop{\mathbb{E}}_{U^1,U^2_{\le N_2}\sim\nu} \mathbb{E}_{j\sim U(N_2)} I(X^1 : X^2_j|X^2_1, \ldots, X^2_{j-1})_{P(\cdot|U^1,U^2_{\le N_2})} \\
&= \mathop{\mathbb{E}}_{U^1,U^2_{\le N_2}\sim\nu} \mathbb{E}_{j\sim U(N_2)} I(X^1 : X^2_j)_{\tilde{P}(\cdot|U^1,U^2_j)},
\end{aligned} \tag{52}$$

where $U(N_2)$ is the uniform distribution over the set $[N_2]$.

Therefore, if $j, U^1, U^2_{\leq N_2}$ are chosen from an ε-SV source ν, we find

$$\begin{aligned}
&\mathop{\mathbb{E}}_{(j,U^1,U^2_{\leq N_2})\sim\nu}\ \mathop{\mathbb{E}}_{X^1,X^2_{<j}\sim P} I(X^1 : X^2_{A_2})_{\tilde{P}(\cdot|U^1,U^2_j)} \\
&\qquad = \mathop{\mathbb{E}}_{(U^1,U^2_{\leq N_2})\sim\nu}\ \mathop{\mathbb{E}}_{j\sim\nu(.|U^1,U^2_{\leq N_2})} \mathbb{E}_{X^1,X^2_{<j}\sim P} I(X^1 : X^2_{A_2})_{\tilde{P}(\cdot|U^1,U^2_j)} \\
&\qquad \leq \left(\frac{1}{2}+\varepsilon\right)^{\log N_2} \mathop{\mathbb{E}}_{(U^1,U^2_{\leq N_2})\sim\nu}\ \mathop{\mathbb{E}}_{j\sim U(N_2)}\ \mathop{\mathbb{E}}_{X^1,X^2_{<j}\sim P} I(X^1 : X^2_j)_{\tilde{P}(\cdot|U^1,U^2_j)} \qquad (53)\\
&\qquad \leq \left(\frac{1}{2}+\varepsilon\right)^{\log N_2} n\log|\Sigma|.
\end{aligned}$$

We now use Pinsker's inequality relating the mutual information and trace distance for any measure Q as

$$I(A : B)_Q \geq \frac{1}{2\ln(2)} \|Q(A,B) - Q(A)\otimes Q(B)\|^2 \qquad (54)$$

with $Q := \tilde{P}(\cdot|U^1, U^2_j)$, and the convexity of $x \mapsto x^2$ to obtain Eq. (50). □

SUPPLEMENTARY NOTE 5: Security proof of Protocol I

In Protocol I, the honest parties and Eve share a no-signaling box $P(x, z|u', w)$, where (x, u') denotes the outputs and inputs of all the honest parties for the n runs of the protocol and (z, w) denotes the output and input of the adversary Eve. The honest parties obtain bits u from the SV source that will serve as inputs to their box, so u' will be set to be equal to u. They also draw further bits t from the SV source to feed together with x into the randomness extractor, obtaining the final output of the protocol

$$s = s(x,t). \qquad (55)$$

Eve has classical information e, which are bits correlated to u, t. The initial box, describing all initial variables and inputs is given by

$$p(x, z, u, t, e|u', w), \qquad (56)$$

which is a family of probability distributions labelled by u' and w (we also denote them by $p_{u',w}(x, z, u, t, e)$). The described setup is illustrated in Supplementary Figure 5.

In notation from Supplementary Lemma 10 on min-entropy sources from Supplementary Note 4, we have $x = (x_1, \ldots, x_n)$, $u = (u_1, \ldots, u_n)$ where every x_k and u_k, for $k \in \{1, \ldots, n\}$, are of the form $\mathbf{x} = (\mathbf{x}^1, \mathbf{x}^2, \mathbf{x}^3, \mathbf{x}^4)$ and $\mathbf{u} = (\mathbf{u}^1, \mathbf{u}^2, \mathbf{u}^3, \mathbf{u}^4)$, respectively.

Now, referring to notation in the assumptions detailed in Supplementary Note 3, for the present protocol, we have $\mathtt{sv}_1 = u$, $\mathtt{sv}_2 = t$, $\mathtt{sv}_E = e$, $\mathtt{out} = x$, $\mathtt{in} = u'$, $\mathtt{in}_E = w$, $\mathtt{out}_E = z$. The assumptions thus read as follows.

- **No-signaling assumptions:**

$$\begin{aligned}
p(x|u', w) &= p(x|u'), & (57)\\
p(z|u', w) &= p(z|w), & (58)\\
p(x_j|u', w, z, u, t, e) &= p(x_j|u'_{\leq j}, w, z, u, t, e) \quad \forall j \in [n]. & (59)
\end{aligned}$$

- **Assumption A1-(I):** The devices do not signal to the SV source, i.e. the distribution of (u, t, e) is independent of the inputs (u', w):

$$\sum_{x,z} p(x, z, u, t, e|u', w) = p(u, t, e) \quad \forall(u, t, e, u', w). \tag{60}$$

- **Assumption A2-(I):** The box is fixed independently of the SV source:

$$p(x, z|u', w, u, t, e) = p(x, z|u', w) \quad \forall(x, z, u, t, e, u', w). \tag{61}$$

- **SV conditions:** The distribution $p(u, t, e)$ satisfies the SV condition (1); in particular, $p(t|u, e)$ satisfies Eq. (1) too.

After inputting u as u' (as is done in the protocol) we obtain

$$p_w(x, z, u, t, e) := p(x, z, u, t, e|u, w). \tag{62}$$

We note that, due to Assmuption A1-(I) (i.e. no-signaling from input u' to the variable u), it is a normalized probability distribution for every w.

Now, for

$$L(x, u) = \frac{1}{n} \sum_{i=1}^{n} B_i(x_i, u_i), \tag{63}$$

we define the sets ACC and ACC_u for acceptance of the protocol as follows

$$\text{ACC} = \{(x, u) : L(x, u) \leq \delta\}, \tag{64}$$

and

$$\text{ACC}_u = \{x : (x, u) \in \text{ACC}\}. \tag{65}$$

Upon acceptance of the protocol, the family of probability distributions (62) is modified to $p_w(x, z, u, t, e|\text{ACC})$.

To quantify the quality of the output s, we will use the universally composable distance defined in Eq. (22), which in this case reads as

$$d_{\text{comp}} = \sum_{s,e} \max_{w} \sum_{z} \left| p_w(s, z, e|\text{ACC}) - \frac{1}{|S|} p_w(z, e|\text{ACC}) \right| \tag{66}$$

with s given by Eq. (55). Here the probability distributions $p_w(s, z, e|\text{ACC}) = \sum_u p_w(s, z, u, e|\text{ACC})$ are computed from probability distributions (62). Actually, in the proofs we will deal with slightly modified distance

$$\tilde{d}_{\text{comp}} := \sum_{s,e} \max_{w} \sum_{z,u} \left| p_w(s, z, u, e|\text{ACC}) - \frac{1}{|S|} p_w(z, u, e|\text{ACC}) \right|. \tag{67}$$

By triangle inequality, we have

$$d_{\text{comp}} \leq \tilde{d}_{\text{comp}} \tag{68}$$

and hence it is enough to bound $\tilde{d}_{\text{comp}}$.

We now define an auxiliary quantity

$$d' := \sum_e p(e|\text{ACC}) \max_w \sum_{z,u} p_w(z,u|e,\text{ACC}) \sum_s \left| p_w(s|z,u,e,\text{ACC}) - \frac{1}{|S|} \right| \tag{69}$$

for any family of probability distributions $\{p_w(x,z,u,t,e)\}$.

Remark 12. *In the last two sections we many times use the following, easy to prove, implication*

$$P(a|b,c,d) = P(a|b) \quad \Rightarrow \quad P(a|b,c,d) = P(a|b,c) = P(a|b,d). \tag{70}$$

where P is an arbitrary probability measure.

From Assumption A1-(I) and A2-(I), as well as no-signaling assumptions (Eqs. (57)-(59)), we find that the distributions $\{p_w(x,z,u,t,e)\}$ satisfy:

$$p_w(x,u) = p(x,u) \quad \text{(implied by Eq. (57) and A1-(I))}, \tag{71}$$
$$p_w(u,t,e) = p(u,t,e) \quad \text{(follows from A1-(I))}, \tag{72}$$
$$\forall_w\, p_w(x,z|u,t,e) = p_w(x,z|u) \quad \text{(follows directly from A2-(I))}, \tag{73}$$
$$\forall_w\, p_w(x,z|u,t,e) = p_w(x,z|u,e) \quad \text{(follows from A2-(I) and Rem. 12)}, \tag{74}$$
$$p_w(x|z,u,t,e) = p_{z,t,e,w}(x|u) \quad \text{is time-ordered no-signaling box (by (59))}, \tag{75}$$
$$p_w(u|z,e) \text{ and } p_w(t|z,u,e) \quad \text{are SV sources (from A2-(I) and SV conditions above)}. \tag{76}$$

Remark 13. *To be precise, the proof of property (76) goes as follows. We know that $p(u,t,e)$ is an SV source. Note that*

$$p_w(u|z) = \frac{p_w(u,z)}{p_w(z)} = \frac{\sum_{t,e} p_w(z,u,t,e)}{p_w(z)} \overset{\text{A2-(II) \& Eq. (58)}}{=} \frac{p_w(z)\sum_{t,e} p_w(u,t,e)}{p_w(z)} \overset{\text{A1-(I)}}{=} \sum_{t,e} p(u,t,e) \tag{77}$$

and hence $p_w(u|z)$ is an SV source. Further, we also obtain

$$p_w(t|z,u,e) = \frac{p_w(z,u,t,e)}{\sum_t p_w(z,u,t,e)} \overset{\text{A2-(I) \& Eq. (58)}}{=} \frac{p_w(z)p_w(u,t,e)}{p_w(z)\sum_t p_w(u,t,e)} = p_w(t|u,e) \tag{78}$$

and therefore, by the fact that $p(t|u,e)$ satisfies the SV condition (1), the distribution $p_w(t|z,u,e)$ also is an SV source.

For each e, let w_e denote Eve's input w and let $p_{w_e}(x,z,u,t|e)$ denote the corresponding distribution that achieves the maximum in Eq.(69). Using the fact that $p_w(e) = p(e)$ and that ACC is a set of (x,u) which obey $p_w(x,u) = p(x,u)$ (from Eq.(71)), we see that the distribution that achieves the maximum takes the form $p(e)p_{w_e}(x,z,u,t|e)$. We now set

$$q(x,z,u,t,e) := p(e)p_{w_e}(x,z,u,t|e). \tag{79}$$

It can be readily seen that this $q(x,z,u,t,e)$ obeys the restrictions:

$$q(x,z|u,t,e) = q(x,z|u), \tag{80}$$
$$q(x,z|u,t,e) = q(x,z|u,e), \tag{81}$$
$$q(x|z,u,t,e) = q_{t,e,z}(x|u) \quad \text{is time ordered no-signaling box}, \tag{82}$$
$$q(u|z,e) \text{ and } q(t|z,u,e) \quad \text{are SV sources}. \tag{83}$$

We can therefore define

$$d := \sum_{z,u,e} q(z,u,e|\text{ACC}) \sum_s \left| q(s|z,u,e,\text{ACC}) - \frac{1}{|S|} \right| \tag{84}$$

and observe that

$$d = d'. \tag{85}$$

We now have

Proposition 14. *For any distribution $p_w(x,z,u,t,e)$, given by Eq. (62), we have*

$$d_{comp} \le |S| d. \tag{86}$$

Proof. This is a consequence of Eq. (68) and the following inequalities

$$\begin{aligned} d_{\text{comp}} \le \tilde{d}_{\text{comp}} &= \sum_{s,e} p(e|\text{ACC}) \max_w \sum_{z,u} p_w(z,u|e,\text{ACC}) \left| p_w(s|u,e,z,\text{ACC}) - \frac{1}{|S|} \right| \\ &\le \sum_{s,e} p(e|\text{ACC}) \max_w \sum_{s',z,u} p(z,u|e,\text{ACC}) \left| p_w(s'|z,u,e,\text{ACC}) - \frac{1}{|S|} \right| \\ &\le |S| \sum_e p(e|\text{ACC}) \max_w \sum_{s,z,u} p_w(z,u|e,\text{ACC}) \left| p_w(s|z,u,e,\text{ACC}) - \frac{1}{|S|} \right| \\ &= |S| d' \overset{(85)}{=} |S| d. \end{aligned} \tag{87}$$

□

We therefore see that we can effectively work with the distribution $q(x,z,u,t,e)$ and d.

Lemma 15. *For any probability distribution $q(x,z,u,t,e)$ satisfying Eqs. (80)-(83) it follows that*

$$q(x|z,u,t,e,ACC) = q(x|z,u,ACC). \tag{88}$$

Proof. For $(x,u) \notin ACC$ the claim holds trivially, since $q(x|z,u,t,e,ACC) = q(x|z,u,e,ACC) = 0$. For $(x,u) \in ACC$, we have

$$\begin{aligned} q(x|z,u,t,e,ACC) &= \frac{q(x, ACC_u|z,u,t,e)}{\sum_{(x,u)\in ACC} q(x,u|z,u,t,e)} \\ &= \frac{q(x|z,u,t,e)}{\sum_{x\in ACC_u} q(x|z,u,t,e)} \\ &\overset{\text{Eq. (80)}}{=} \frac{q(x|z,u)}{\sum_{x\in ACC_u} q(x|z,u)} \\ &= q(x|z,u,ACC), \end{aligned} \tag{89}$$

which proves the claim. □

Lemma 16. *Consider the measure $q(x,z,u,t,e)$ satisfying conditions given by Eqs. (80)-(83). Let $\delta, \delta_{Az} > 0$ be constants and define the set*

$$A^{\delta_{Az}} := \{(z,u,e) : Pr_{\sim q(x|z,u,e)}(\bar{L} \ge L + \delta_{Az}) \le \epsilon_{Az}\}, \tag{90}$$

where $\epsilon_{Az} := 2e^{-\frac{1}{4}\delta_{Az}^2 n}$ *and*

$$L(x,u) := \frac{1}{n}\sum_{i=1}^{n} B_i(x_i,u_i), \tag{91}$$

$$\overline{L}(x,z,u,e) := \frac{1}{n}\sum_{i=1}^{n} \mathbb{E}_{q(x_i,u_i|x_{<i},z,u_{<i},e)} B_i(x_i,u_i). \tag{92}$$

Let $(z,u,e) \in A^{\delta_{Az}}$*. Then, for arbitrary* $x \in ACC_u$*, we obtain*

$$q(x|z,u,e) \le \max\{\epsilon_{Az}, \gamma^{\mu n}\}, \tag{93}$$

where

$$\mu := 1 - \sqrt{\delta + \delta_{Az}}, \qquad \gamma = \frac{1}{3}\left(1 + 2\frac{\sqrt{\delta+\delta_{Az}}}{(\frac{1}{2}-\epsilon)^4}\right). \tag{94}$$

Proof. Let $(z,u,e) \in A^{\delta_{Az}}$ and $x \in \mathrm{ACC}_u$. We further define two sets:

$$X_{\text{good}}^{(z,u,e)} = \{x : |L(x,u) - \overline{L}(x,z,u,e)| \le \delta_{Az}\} \tag{95}$$

and

$$X_{\text{bad}}^{(z,u,e)} = \left(X_{\text{good}}^{(z,u,e)}\right)^c. \tag{96}$$

Note that, for $(z,u,e) \in A^{\delta_{Az}}$ and $x \in X_{\text{bad}}^{(z,u,e)}$, we have

$$Pr_{\sim q(x|z,u,e)}(|L(x,u) - \overline{L}(x,z,u,e)| > \delta_{Az}) \le \epsilon_{Az}. \tag{97}$$

Hence, for $x \in X_{\text{bad}}^{(z,u,e)}$,

$$q(x|z,u,e) \le \epsilon_{Az}. \tag{98}$$

Let us now analyze the case, when $x \in X_{\text{good}}^{(z,u,e)}$. Since $x \in \mathrm{ACC}_u$, which implies $(x,u) \in \mathrm{ACC}$, we have $L(x,u) \le \delta$. Further, for $x \in X_{\text{good}}^{(z,u,e)}$, we obtain

$$\bar{L}(x,z,u,e) \le \delta + \delta_{Az}. \tag{99}$$

Recall that

$$\bar{B}_i = \mathbb{E}_{\sim q(x_i,u_i|x_{<i},z,u_{<i},e)}(B_i), \tag{100}$$

where $u_{<i}$ and $x_{<i}$ are the components of u and x respectively. Following Supplementary Lemma 7, we know that, for μn positions i, where $\mu := (1 - \sqrt{\delta + \delta_{Az}})$, we get

$$\bar{B}_i \le \sqrt{\delta + \delta_{Az}}. \tag{101}$$

and $\bar{B}_i$ plays the role of $\bar{B}^{SV}$ here. Then, by Supplementary Lemma 8, for μn positions i there holds

$$q_{x_{<i},z,u_{<i},e}(x_i|u_i) \le \gamma, \tag{102}$$

for any measurement outcome x_i and setting u_i. Applying Supplementary Lemma 10, we get

$$q(x|z,u,e) \le \gamma^{\mu n}. \tag{103}$$

Then, due to Eqs. (98) and (103), the proof is completed. □

Proposition 17. *Given that the probability distribution $q(x,z,u,e)$ satisfies conditions (80)-(83), we have*

$$Pr_{\sim q(z,u,e|ACC)}\left(\max_x q(x|z,u,e,ACC) \le \sqrt{\frac{\delta_1}{q(ACC)}}\right) \ge 1-\sqrt{\frac{\delta_1}{q(ACC)}}. \tag{104}$$

where

$$\delta_1 = \gamma^{\mu n} + 2\epsilon_{Az}. \tag{105}$$

Proof. Assume first that $(z,u,e) \in A^{\delta_{Az}}$. We then have

$$\max_x q(x|z,u,e,\mathrm{ACC}) = \max_x \frac{q(x,\mathrm{ACC}|z,u,e)}{q(\mathrm{ACC}|z,u,e)} \tag{106}$$

$$= \frac{\max_{x\in \mathrm{ACC}_u} q(x|z,u,e)}{q(\mathrm{ACC}|z,u,e)}$$

$$\overset{\text{Supplementary Lemma 16}}{\le} \frac{\max\{\epsilon_{Az},\gamma^{\mu n}\}}{q(\mathrm{ACC}|z,u,e)}. \tag{107}$$

We now consider

$$\sum_{z,u,e} q(z,u,e|\mathrm{ACC}) \max_x q(x|z,u,e,\mathrm{ACC})$$
$$= \sum_{(z,u,e)\in A^{\delta_e}} q(z,u,e|\mathrm{ACC}) \max_x q(x|z,u,e,\mathrm{ACC}) + \sum_{(z,u,e)\notin A^{\delta_e}} q(z,u,e|\mathrm{ACC}) \max_x q(x|z,u,e,\mathrm{ACC}). \tag{108}$$

We bound the first terms as follows:

$$\sum_{(z,u,e)\in A^{\delta_{Az}}} q(z,u,e|\mathrm{ACC}) \max_x q(x|z,u,e,\mathrm{ACC}) \overset{\text{Eq. (106)}}{\le} \sum_{(z,u,e)\in A^{\delta_{Az}}} q(z,u,e|\mathrm{ACC}) \frac{\max\{\epsilon_{Az},\gamma^{\mu n}\}}{q(\mathrm{ACC}|z,u,e)}$$
$$\le \max\{\epsilon_{Az},\gamma^{\mu n}\} \sum_{(z,u,e)} \frac{q(z,u,e)}{q(\mathrm{ACC})}$$
$$= \frac{\max\{\epsilon_{Az},\gamma^{\mu n}\}}{q(\mathrm{ACC})}. \tag{109}$$

Let us now apply Supplementary Lemma 5, taking $W_0 = (e,z)$, $W_i = (x_i,u_i)$, for $i = 1,\dots,n$, and B_i, given by Eq. (7). We obtain $q(A^{\delta_{Az}}) \ge 1-\epsilon_{Az}$. Thus, the second term is bounded as follows

$$\sum_{(z,u,e)\notin A^{\delta_{Az}}} q(z,u,e|\mathrm{ACC}) \max_x q(x|z,u,e,\mathrm{ACC}) \le \sum_{(z,u,e)\notin A^{\delta_{Az}}} q(z,u,e|\mathrm{ACC})$$
$$\le \sum_{(z,u,e)\notin A^{\delta_{Az}}} \frac{q(z,u,e)}{q(\mathrm{ACC})}$$
$$\overset{\text{Supplementary Lemma 5}}{\le} \frac{\epsilon_{Az}}{q(\mathrm{ACC})}. \tag{110}$$

Altogether we find

$$\sum_{z,u,e} q(z,u,e|\mathrm{ACC}) \max_x q(x|z,u,e,\mathrm{ACC}) \le \frac{\max\{\epsilon_{Az},\gamma^{\mu n}\}}{q(\mathrm{ACC})} + \frac{\epsilon_{Az}}{q(\mathrm{ACC})} \le \frac{\gamma^{\mu n}+2\epsilon_{Az}}{q(\mathrm{ACC})}.$$

Applying Markov inequality and setting $\delta_1 = \gamma^{\mu n} + 2\epsilon_{Az}$ completes the proof. □

The main theorem of this section is the following:

Theorem 18. *Let n denote the number of runs in Protocol I and suppose we are given $\epsilon > 0$. Set $\delta > 0$ such that*

$$\frac{1}{3}\left(1+2\frac{\sqrt{2\delta}}{(\frac{1}{2}-\epsilon)^4}\right) < 1 \tag{111}$$

(see Supplementary Figure 6 trade-off between δ and ϵ). Then for any probability distribution $p_w(x,z,u,t,e)$ satisfying Eqs. (71)-(76) *there exists a non-explicit extractor $s(x,t)$ with $|S| = 2^{\Omega(n^{1/2})}$ values, such that*

$$d_{comp} \cdot p(ACC) \leq 2^{\Omega(n^{1/2})} \tag{112}$$

where d_{comp} is given Eq. (66). *Alternatively, one can use an explicit extractor $s'(x,t)$ producing a single bit of randomness with*

$$d_{comp} \cdot p(ACC) \leq 2^{-\Omega(n^{1/(2C)})}, \tag{113}$$

for some constant C.

Remark 19. *Note, that due to first condition of* (71), *$p_w(ACC)$ does not depend on w, hence we could have written just $p(ACC)$ in the theorem. Moreover, we even have $q(ACC) = p(ACC)$.*

Proof. Let $\delta > 0$, satisfying Eq. (111), be given. Set $\delta_{Az} = \delta$ so that $\epsilon_{Az} = 2e^{-\frac{1}{4}\delta_{Az}^2 n} = 2^{-\Omega(n)}$. Then $\mu \geq 1 - \sqrt{2\delta}$ and $\gamma < 1$. Now, let $\eta = \sqrt{\gamma^{\mu n} + 2\epsilon_{Az}}$, so that $\eta = 2^{-\Omega(n)}$. We shall now consider distribution q given by Eq. (79) and distance d of (84). Suppose first that $q(\text{ACC}) \leq \eta$ and let us consider

$$d = \sum_{z,u,e} q(z,u,e|\text{ACC}) \sum_{s=1}^{|S|} \left| q(s|z,u,e,\text{ACC}) - \frac{1}{|S|} \right|. \tag{114}$$

Then, since by definition $d \leq 2$, we obtain $d \cdot q(\text{ACC}) \leq 2^{-\Omega(n)}$. Now, suppose in turn, that $q(\text{ACC}) \geq \eta$. Then, from Supplementary Proposition 17 we get

$$Pr_{\sim q(z,u,e|\text{ACC})}\left(\max_x q(x|z,u,e,\text{ACC}) \leq \sqrt{\eta}\right) \geq 1 - \sqrt{\eta}. \tag{115}$$

Take the set Good $= \{(z,u,e) : \max_x q(x|z,u,e,\text{ACC}) \leq \sqrt{\eta}\}$. Then for $(z,u,e) \in$ Good, we have

$$H_{\min}\left(q(x|z,u,e,\text{ACC})\right) \geq cn, \tag{116}$$

for some constant $c > 0$. We now consider the total probability distribution $q(x,z,u,t,e)$. From Eq. (81), we have that conditioned on (z,u,e) the random variables t and x are independent

$$q(x,t|z,u,e) = q(x|z,u,e)q(t|z,u,e). \tag{117}$$

Due to Supplementary Lemma 15, we further obtain

$$q(x,t|z,u,e,\text{ACC}) = q(x|z,u,e,\text{ACC})q(t|z,u,e,\text{ACC}) \tag{118}$$

Moreover, by assumption (i.e. Eq. (83)) $q(t|z,u,e)$ obeys the SV source conditions. Hence, if we show that $q(t|z,u,e,\mathrm{ACC}) = q(t|z,u,e)$, then $q(t|z,u,e,\mathrm{ACC})$ obeys the SV source conditions as well. Note that

$$q(t|z,u,e,\mathrm{ACC}) = \frac{\sum_{x\in\mathrm{ACC}_u} q(t,x|z,u,e)}{\sum_{x\in\mathrm{ACC}_u} q(x|z,u,e)} \overset{\text{Eq.(117)}}{=} q(t|z,u,e). \tag{119}$$

Therefore $q(t|z,u,e,\mathrm{ACC})$ is an min-entropy source and we have

$$H_{\min}(q(t|z,u,e,\mathrm{ACC})) = c'n \tag{120}$$

where c' is a constant depending only on ϵ. Thus $q(x,t|z,u,e,\mathrm{ACC})$ is a product of two min-entropy sources. By the application of the non-explicit extractor of Supplementary Lemma 3 part (i) we obtain the output s with

$$\sum_s \left| q(s|z,u,e,\mathrm{ACC}) - \frac{1}{|S|} \right| \le 2^{-\Omega(n)}. \tag{121}$$

For $(z,u,e) \notin \mathrm{Good}$, we use $\sum_s \left| q(s|z,u,e,\mathrm{ACC}) - \frac{1}{|S|} \right| \le 2$ and obtain

$$\begin{aligned} d \;&\le \sum_{(z,u,e)\in\mathrm{Good}} q(z,u,e|\mathrm{ACC}) 2^{-\Omega(n)} + \sum_{(z,u,e)\notin\mathrm{Good}} 2q(z,u,e|\mathrm{ACC}) \\ &\overset{\text{Eq. (115)}}{\le} 2^{-\Omega(n)} + 2\sqrt{\eta} = 2^{-\Omega(n)}. \end{aligned} \tag{122}$$

(recall we have set $\eta = 2^{-\Omega(n)}$). We thus obtain that

$$d \cdot q(\mathrm{ACC}) \le 2^{-\Omega(n)}. \tag{123}$$

By definition of q (see also Supplementary Remark 19), we have $q(\mathrm{ACC}) = p(\mathrm{ACC})$, and from Supplementary Proposition 14 we know that $d_{\mathrm{comp}} \le |S| d$. So choosing $|S| = 2^{\Omega(n^{1/2})}$ the claim follows. Alternatively, in Eq. (121) we can use the explicit extractor of Supplementary Lemma 3 part (iii) to obtain the single bit output s' with

$$\sum_{s'} \left| q(s'|z,u,e,\mathrm{ACC}) - \frac{1}{2} \right| \le 2^{-\Omega(n^{1/(2C)})}, \tag{124}$$

for some constant C. In this case, we obtain simply $d_{\mathrm{comp}} \cdot q(\mathrm{ACC}) \le 2^{-\Omega(n^{1/(2C)})}$. □

SUPPLEMENTARY NOTE 6: Security proof of Protocol II

Protocol II considers the situation where the honest parties have two no-signaling devices, and share with the adversary Eve a no-signaling box $\{p(x,y^1,\dots,y^N,z|u',v'^1,\dots,v'^N,w)\}$. We work with the probability distribution $p(x,y^1,\dots y^N,z,u,v^1,\dots,v^N,t,j,e,u',v'^1,\dots v'^N,w)$. Here we consider n uses of the first device with $u'(=u'_1,\dots,u'_n)$ denoting the inputs for these, as well as N blocks of the second device (each with n uses) with $v'^1,\dots,v'^N$ denoting the inputs for this device (note $v'^k = v'^k_1,\dots,v'^k_n$). The honest parties draw $u,v^1,\dots,v^N$ as well as the bit strings j and t from the SV source. The parties input u to the first device ($u' = u$) and $v^1,\dots,v^j$ to the second device ($v'^k = v^k$ for $k \in [j]$). They obtain the corresponding outputs x from the first device, and $y^1,\dots,y^j$ from the second device. The remaining variables $y^{j+1},\dots,y^N$ we define

to be zero. The adversary Eve holds the bit string e which is her classical information about the bits drawn from the SV source. Her input is denoted as w with corresponding output z. To avoid cumbersome notation, we will use the shorthand $v'(v)$ to denote $v'^1, \ldots, v'^N$ $(v^1, \ldots, v^N)$ as well as $y = y^1, \ldots, y^N$, where there is no possibility of confusion. Now the initial box describing the initial variables and inputs is given by $p(x, y, z, u, v, t, j, e|u', v', w)$, a family of probability distributions labeled by u', v', w. The final output s of the protocol the honest parties compute as a function of x, y^j and t

$$s = s(x, y^j, t). \tag{125}$$

Referring to notation in Supplementary Note 3, for the present protocol, we have $\texttt{sv}_1 = (u, v)$, $\texttt{sv}_2 = (t, j)$, $\texttt{sv}_E = e$, $\texttt{out} = (x, y)$, $\texttt{in} = (u', v')$, $\texttt{in}_E = w$, $\texttt{out}_E = z$. The assumptions of Supplementary Note 3 thus read as follows.

- **No-signaling assumptions:**
  We have full no-signaling between all parties and devices (see Supplementary Figure 7), i.e.

$$\begin{aligned} p(x, y|u', v', w) &= p(x, y|u', v') \text{ (no-signaling from Eve to honest parties)}, & (126)\\ p(z|u', v', w) &= p(z|w) \text{ (no-signaling from honest parties to Eve)}, & (127)\\ p(x, z|u', v', w) &= p(x, z|u', w), & (128)\\ p(y, z|u', v', w) &= p(y, z|v', w). & (129) \end{aligned}$$

  We also assume the following time ordered no-signaling conditions (see Supplementary Figure 7).

$$\begin{aligned} p(x_k|u', v', w, z, u, v, t, j, e) &= p(x_k|u'_{\leq k}, v', w, z, u, v, t, j, e) \quad \forall k \in [n], & (130)\\ p(y_k|u', v', w, z, u, v, t, j, e) &= p(y_k|u', v'_{\leq k}, w, z, u, v, t, j, e) \quad \forall j \in [n]. & (131) \end{aligned}$$

- **Assumption A1-(II):** The devices do not signal to the SV source, i.e., the distribution of (u, v, t, j, e) is independent of the inputs (u', v', w):

$$p(u, v, t, j, e|u', v', w) = p(u, v, t, j, e) \quad \forall (u, v, t, j, e, u', v', w). \tag{132}$$

- **Assumption A2-(II):** The form of the box is fixed independently of the SV source:

$$p(x, y, z|u', v', w, u, v, t, j, e) = p(x, y, z|u', v', w) \quad \forall (x, y, z, u, v, t, j, e, u', v', w). \tag{133}$$

- **SV conditions:** The distribution $p(u, v, t, j, e)$ satisfies an SV condition (1); in particular, $p(t|u, v, j, e)$ satisfies Eq. (1) too.

Remark 20. *Note that*

$$p(x|z, u', v', w) = \frac{p(x, z|u', v', w)}{p(z|u', v', w)} \overset{Eq.(128)}{=} \frac{p(x, z|u', w)}{p(z|u', v', w)} \overset{Eqs.(127),(70)}{=} \frac{p(x, z|u', w)}{p(z|u', w)} = p(x|z, u', w) \tag{134}$$

and similary we may show that

$$p(y|z, u', v', w) = p(y|z, v', w). \tag{135}$$

After the honest parties input $u' = u, v' = v$ in the protocol, we work with the distribution

$$p_w(x, y, z, u, v, t, j, e) := p(x, y, z, u, v, t, j, e|w) \tag{136}$$

Assumption A1-(II) ensures that this is a normalized probability distribution. The setup is shown in Supplementary Figure 7.

We will use the further shorthand notation $R_j := (y \setminus y^j, v \setminus v^j)$ and $M_j := (R_j, j)$. Further, we define the sets of acceptance as follows

$$\mathrm{ACC}^1 := \{(x, u) : L(x, u) \leq \delta\}, \tag{137}$$
$$\mathrm{ACC}^j := \{(y^j, v^j) : L(y^j, v^j) \leq \delta\}, \tag{138}$$
$$\mathrm{ACC} := \mathrm{ACC}^1 \cap \mathrm{ACC}^j, \tag{139}$$

where $L(x, u) = \frac{1}{n}\sum_{i=1}^n B_i(x_i, u_i)$ and $L(y^j, v^j) = \frac{1}{n}\sum_{i=1}^n B_i(y_i^j, v_i^j)$. We also define the u-cut of the set ACC^1 as $\mathrm{ACC}^1_u := \{x : (x, u) \in \mathrm{ACC}^1\}$ and the v^j-cut of the set ACC^j as $\mathrm{ACC}^j_{v^j} := \{y^j : (y^j, v^j) \in \mathrm{ACC}^j\}$.

To quantify the quality of the output we will use the universally composable distance defined in Eq. (22), which is of the same form as the distance in Eq. (66) used in Protocol I

$$d^{\mathrm{II}}_{\mathrm{comp}} := \sum_{s,e} \max_w \sum_z \left| p_w(s, z, e|\mathrm{ACC}) - \frac{1}{|S|} p_w(z, e|\mathrm{ACC}) \right| \tag{140}$$

with s given by Eq. (125). The probability distribution $p_w(s, z, e|\mathrm{ACC})$ is computed from the probability distributions $p_w(x, y, z, u, v, t, j, e)$ given by Eq. (136). Actually, in the proofs we will deal with slightly modified distance

$$\tilde{d}^{\mathrm{II}}_{\mathrm{comp}} := \sum_{s,e} \max_w \sum_{z,u,v^j,M_j} \left| p_w(s, z, u, v^j, M_j, e|\mathrm{ACC}) - \frac{1}{|S|} p_w(z, u, v^j, M_j, e|\mathrm{ACC}) \right|, \tag{141}$$

By triangle inequality, we have

$$d^{\mathrm{II}}_{\mathrm{comp}} \leq \tilde{d}^{\mathrm{II}}_{\mathrm{comp}} \tag{142}$$

and hence it is enough to bound $\tilde{d}^{\mathrm{II}}_{\mathrm{comp}}$.

As in Protocol I, we now define the distance quantities d' and d to see that we can work with a probability distribution without w. Let us first define the analogous quantity d'_{II} for Protocol II

$$d'_{\mathrm{II}} := \sum_e p(e|\mathrm{ACC}) \max_w \sum_{z,u,v^j,M_j} p_w(z, u, v^j, M_j|e, \mathrm{ACC}) \sum_s \left| p_w(s|z, u, v^j, M_j, e, \mathrm{ACC}) - \frac{1}{|S|} \right| \tag{143}$$

where $p_w(s, z, u, v^j, M_j, e|\mathrm{ACC})$ is computed from any family of probability distributions

$\{p_w(x,y,z,u,v,t,j,e)\}$ satisfying

$$p_w(x,y,u,v) = p(x,y,u,v) \quad \text{(by Eq. (144) and A1-(II))}, \tag{144}$$
$$p_w(u,v,t,j,e) = p(u,v,t,j,e) \quad \text{(follows from A1-(II))}, \tag{145}$$
$$\forall_w\ p_w(x,y,z|u,v,t,j,e) = p_w(x,y,z|u,v) \quad \text{(follows directly from A2-(II))}, \tag{146}$$
$$\forall_w\ p_w(x,y,z|u,v,t,j,e) = p_w(x,y,z|u,v,j,e) \quad \text{(by A2-(II) and Rem. 12)}, \tag{147}$$
$$p_w(x|z,u,v,t,j,e) = p_w(x|z,u,t,j,e) \quad \text{(by A2-(II) and Eq. (134))}, \tag{148}$$
$$p_w(y|z,u,v,t,j,e) = p_w(y|z,v,t,j,e) \quad \text{(by A2-(II) and Eq. (135))}, \tag{149}$$
$$p_w(x|z,u,v,t,j,e) = p_{w,z,v,t,j,e}(x|u) \text{ and } p_w(y|z,u,v,t,j,e) = p_{w,z,u,t,j,e}(y|v)$$
$$\text{are time-ordered no-signaling (tons) boxes by Eqs. (130) and (131)}, \tag{150}$$
$$p_w(u|z,e),\ p_w(v|z,u,e),\ p_w(j|z,u,v,e) \text{ and } p_w(t|z,u,v,j,e) \text{ are SV sources (by A2-(II),}$$
$$\text{Rem. (12) and SV conditions - the proof goes in the same manner as in Rem. 13)}. \tag{151}$$

For each e, let w_e denote Eve's input w and let $p_{w_e}(x,y,z,u,v,t,j|e)$ denote the corresponding distribution that achieves the maximum in Eq. (143). Using the fact that $p_w(e) = p(e)$ and that ACC is a set of (x,y,u,v) which obey $p_w(x,y,u,v) = p(x,y,u,v)$ (from Eq. (144)), we see that the distribution that achieves the maximum takes the form $p(e)p_{w_e}(x,y,z,u,v,t,j|e)$. We now set

$$q(x,y,z,u,v,t,j,e) := p(e)p_{w_e}(x,y,z,u,v,t,j|e). \tag{152}$$

It can be readily seen that this $q(x,y,z,u,v,t,j,e)$ obeys the restrictions:

$$q(x,y,z|u,v,t,j,e) = q(x,y,z|u,v), \tag{153}$$
$$q(x,y,z|u,v,t,j,e) = q(x,y,z|u,v,j,e), \tag{154}$$
$$q(x|z,u,v,t,j,e) = q(x|z,u,t,j,e) \text{ and } q(y|z,u,v,t,j,e) = q(y|z,v,t,j,e), \tag{155}$$
$$q(x|z,u,v,t,j,e) = q_{z,v,t,j,e}(x|u) \text{ and } q(y|z,u,v,t,j,e) = q_{z,u,t,j,e}(y|v) \quad \text{are tons boxes}, \tag{156}$$
$$q(u|z,e), q(v|z,u,e), q(j|z,u,v,e) \text{ and } q(t|z,u,v,j,e) \quad \text{are SV sources}. \tag{157}$$

We can therefore define

$$d_{\text{II}} := \sum_{z,u,v^j,M_j,e} q(z,u,v^j,M_j,e|\text{ACC}) \sum_s \left| q(s|z,u,v^j,M_j,e,\text{ACC}) - \frac{1}{|S|} \right|. \tag{158}$$

and observe that $d_{\text{II}} = d'_{\text{II}}$.

As for protocol I, the distance quantity $d^{\text{II}}_{\text{comp}}$ is bounded as in the following proposition.

Proposition 21. *For any distribution $p_w(x,y,z,u,v,t,j,e)$ given by Eq. (136) we have*

$$d^{II}_{comp} \le |S| d_{II}. \tag{159}$$

Proof. The proof is analogous to that of Supplementary Lemma 14 with the substitution $u \to (u, v^j M_j)$. □

We therefore see that we can effectively work with the distribution $q(x,y,z,u,v,t,j,e)$ and d_{II}. We now define the set $A^{\delta_{Az}}$ for which Azuma estimation works, as follows

$$A^{\delta_{Az},1} = \{(z,u,v^j,M_j,e) : Pr_{\sim q(x|z,u,v^j,M_j,e)} \left(\overline{L}(x,z,u,M_j,e) \le L(x,u) + \delta_{Az}\right) \ge 1 - \epsilon_{Az}\},$$
$$A^{\delta_{Az},j} = \{(z,u,v^j,M_j,e) : Pr_{\sim q(y^j|z,u,v^j,M_j,e)} \left(\overline{L}(y^j,z,v^j,M_j,e) \le L(y^j,v^j) + \delta_{Az}\right) \ge 1 - \epsilon_{Az}\},$$
$$A^{\delta_{Az}} = A^{\delta_{Az},1} \cap A^{\delta_{Az},j}, \tag{160}$$

where $\epsilon_{Az} := 2e^{-\frac{1}{4}\delta_{Az}^2 n}$ and $\overline{L}$ is of the form

$$\overline{L}(x,z,u,M_j,e) := \frac{1}{n}\sum_{i=1}^{n}\mathbb{E}_{\sim q(x_i,u_i|x_{<i},z,u_{<i},M_j,e)}B_i(x_i,u_i), \tag{161}$$

$$\overline{L}(y^j,z,v^j,M_j,e) := \frac{1}{n}\sum_{i=1}^{n}\mathbb{E}_{\sim q(y_i^j,v_i^j|z,y_{<i}^j,v_{<i}^j,M_j,e)}B_i(y_i^j,v_i^j), \tag{162}$$

where u_i and v_i^j are distributed according to the measure ν from an SV source. Further, we define

$$S^{\xi} = \left\{(z,u,v^j,M_j,e) : Pr_{\sim q(x,y^j|z,u,v^j,M_j,e)}\left(\mathrm{ACC}_u^1 \cap \mathrm{ACC}_{v^j}^j\right) \geq \xi\right\}. \tag{163}$$

and

$$D_{\epsilon_{deF}} := \{(z,u,v^j,M_j,e) : \|q(x,y^j|z,u,v^j,M_j,e) - q(x|z,u,v^j,M_j,e)\otimes q(y^j|z,u,v^j,M_j,e)\| \leq \epsilon_{deF}\}. \tag{164}$$

We note that for any fixed M_j, the boxes corresponding to both blocks $q(x|z,u,M_j,e)$ and $q(y^j|z,v^j,M_j,e)$ are valid time ordered no-signaling boxes, as it was in the single device scenario. Indeed, for the first quantity we have

$$q(x|z,u,M_j,e) = q(x|z,y\setminus y^j,u,v\setminus v^j,j,e) = q(x|z,y^{<j},u,v\setminus v^j,j,e), \tag{165}$$

where we use $y^k = 0$ for $k > j$ and in this distribution the inputs and outputs of the second device (that does not signal to the first) $v\setminus v^j$ and $y^{<j}$ are just labels for the distribution.

Regarding the second device, we have

$$q(y^j|z,v^j,M_j,e) = q(y^j|y\setminus y^j,z,v^j,v\setminus v^j,j,e) = q(y^j|y^{<j},z,v^j,v\setminus v^j,j,e), \tag{166}$$

where we used the fact that $y^k = 0$ for $k > j$. Due to the assumption that, conditioned on the past, the box is still time-ordered no-signaling and because $v^{>j}$ are just random variables from SV source, we have again that the latter distribution is a time-ordered no-signaling box and $v\setminus v^j$, $y^{<j}$ and j,z,e are just labels. We now observe the analogue of Supplementary Lemma 16.

Proposition 22. *Consider the measure $q(x,y^j,z,u,v^j,M_j,e)$ satisfying conditions (153)-(157). Let $\delta,\delta_{Az} > 0$ be constants and let $(z,u,v^j,M_j,e) \in A^{\delta_{Az}}$. Then, for arbitrary $x \in ACC_u^1$, we have*

$$q(x|z,u,M_j,e) \leq \max\{\gamma^{\mu n},\epsilon_{Az}\}, \tag{167}$$

and for arbitrary $y^j \in ACC_{v^j}^j$,

$$q(y^j|z,v^j,M_j,e) \leq \max\{\gamma^{\mu n},\epsilon_{Az}\}, \tag{168}$$

where

$$\mu := 1 - \sqrt{\delta+\delta_{Az}}, \qquad \gamma = \frac{1}{3}\left(1 + 2\frac{\sqrt{\delta+\delta_{Az}}}{(\frac{1}{2}-\epsilon)^4}\right). \tag{169}$$

Proof. The proof is analogous to that of Supplementary Lemma 16 with the direct substitution $u \to (u,v^j,M_j)$ and noting that, by no-signaling (i.e. Eq. (155)) and Supplementary Remark 12, we have $q(y^j|z,u,v^j,M_j,e) = q(y^j|z,v^j,M_j,e)$ and $q(x|z,u,v^j,M_j,e) = q(x|z,u,M_j,e)$. □

We now also have the analogue of Supplementary Proposition 17.

Proposition 23. *Fix arbitrary* $\delta, \delta_{Az} > 0$ *and consider the measure* $q(x, y^j, z, u, v^j, M_j, e)$ *that satisfies conditions (153)-(157). We have*

$$Pr_{\sim q(z,u,v^j,M_j,e|ACC^1)}\left(\max_x q(x|z,u,v^j,M_j,e,ACC^1) \leq \sqrt{\frac{\delta_1}{q(ACC^1)}}\right) \geq 1 - \sqrt{\frac{\delta_1}{q(ACC^1)}}, \quad (170)$$

$$Pr_{\sim q(z,u,v^j,M_j,e|ACC^j)}\left(\max_{y^j} q(y^j|z,u,v^j,M_j,e,ACC^j) \leq \sqrt{\frac{\delta_1}{q(ACC^j)}}\right) \geq 1 - \sqrt{\frac{\delta_1}{q(ACC^j)}}, \quad (171)$$

where

$$q(ACC^1) = Pr_{\sim q(x,z,u,v^j,M_j,e)}(ACC^1), \quad q(ACC^j) = Pr_{\sim q(y^j,z,u,v^j,M_j,e)}(ACC^j) \quad (172)$$

and

$$\delta_1 = \gamma^{\mu n} + 2\epsilon_{Az}. \quad (173)$$

Proof. The proof is analogous to that of Supplementary Proposition 17 if we substitute $u \to (u, v^j M_j)$ and ACC $\to$ ACC^1 for Eq.(170) and ACC $\to$ ACC^j for Eq.(171). We also use Supplementary Lemma 5 with $W_i = (u_i, x_i)$ for $i \geq 1$ and $W_0 = (z, v^j, M_j, e)$ for Eq.(170), and with $W_i = (v_i^j, y_i^j)$ for $i \geq 1$ and $W_0 = (z, u, M_j, e)$, for Eq.(171). □

Here we shall prove a proposition that has no analogue in the security proof for Protocol I. It says that, if the original distribution is close to product (due to our deFinetti-type result), then also the distribution conditioned upon acceptance will be close to product of distributions.

Proposition 24. *For arbitrary* $\epsilon_{deF} > 0$, $\xi \geq 2\epsilon_{deF}$ *and* $M \equiv (z, u, v^j, M_j, e) \in S^\xi$ *suppose*

$$\left\| q(x, y^j|M) - q(x|M) \otimes q(y^j|M) \right\| \leq \epsilon_{deF}. \quad (174)$$

Then we have

$$\left\| q(x, y^j|M, ACC) - q(x|M, ACC^1) \otimes q(y^j|M, ACC^j) \right\| \leq \frac{3\epsilon_{deF}}{\xi^2}. \quad (175)$$

Proof. Since ACC $=$ ACC$^1 \cap$ ACC^j, Eq. (174) implies

$$\left| q(\text{ACC}|M) - q(\text{ACC}^1|M) q(\text{ACC}^j|M) \right| \leq \epsilon_{deF}. \quad (176)$$

Hence

$$\left\| q(x, y^j|M, \text{ACC}) - q(x|M, \text{ACC}^1) \otimes q(y^j|M, \text{ACC}^j) \right\|$$
$$\leq \max_{\pm} \left\| \frac{q(x, y^j, \text{ACC}|M)}{q(\text{ACC}|M)} - \frac{q(x, \text{ACC}^1|M) \otimes q(y^j, \text{ACC}^j|M)}{q(\text{ACC}|M)(1 \pm \kappa)} \right\| \quad (177)$$

where $\kappa = \frac{\epsilon_{deF}}{q(\text{ACC}|M)}$. For $M \equiv (z, u, v^j, M_j, e) \in S^\xi$, we have by definition of S^ξ in Eq. (163) that $q(\text{ACC}|M) \geq \xi$, so that $\kappa \leq \frac{\epsilon_{deF}}{\xi} \leq \frac{1}{2}$ for $\xi \geq 2\epsilon_{deF}$. Sandwiching the above with $\frac{q(x,\text{ACC}^1|M) \otimes q(y^j,\text{ACC}^j|M)}{q(\text{ACC}|M)}$ and using triangle inequality we get

$$\begin{aligned}
&\left\| q(x, y^j|M, \text{ACC}) - q(x|M, \text{ACC}^1) \otimes q(y^j|M, \text{ACC}^j) \right\| \\
&\leq \left\| \frac{q(x, y^j, \text{ACC}|M)}{q(\text{ACC}|M)} - \frac{q(x, \text{ACC}^1|M) \otimes q(y^j, \text{ACC}^j|M)}{q(\text{ACC}|M)} \right\| \\
&+ \left\| \frac{q(x, \text{ACC}^1|M) \otimes q(y^j, \text{ACC}^j|M)}{q(\text{ACC}|M)} \left(1 - \frac{1}{1 \pm \kappa}\right) \right\| \qquad (178)
\end{aligned}$$

Now we can bound the first term on the right hand side of Eq. (178) as follows

$$\begin{aligned}
&\left\| \frac{q(x, y^j, \text{ACC}|M)}{q(\text{ACC}|M)} - \frac{q(x, \text{ACC}^1|M) \otimes q(y^j, \text{ACC}^j|M)}{q(\text{ACC}|M)} \right\| \\
&\leq \frac{1}{q(\text{ACC}|M)} \left\| q(x, y^j|M) - q(x|M) \otimes q(y^j|M) \right\| \overset{\text{Eq. (174)}}{\leq} \frac{\epsilon_{deF}}{q(\text{ACC}|M)}, \qquad (179)
\end{aligned}$$

since projecting onto ACC is a trace non-increasing channel and hence cannot increase trace norm. The second term on the right hand side of Eq. (178) is simply bounded as

$$\left\| \frac{q(x, \text{ACC}^1|M) \otimes q(y^j, \text{ACC}^j|M)}{q(\text{ACC}|M)} \left(1 - \frac{1}{1 \pm \kappa}\right) \right\| \leq \frac{1}{q(\text{ACC}|M)} \left| \frac{\kappa}{1 - \kappa} \right| \leq \frac{2\kappa}{q(\text{ACC}|M)}, \qquad (180)$$

where $\kappa \leq \frac{\epsilon_{deF}}{\xi} \leq \frac{1}{2}$. Inserting these into (178) and using $1 \geq q(\text{ACC}|M) \geq \xi$ for $M \in S^\xi$, we get

$$\left\| q(x, y^j|M, \text{ACC}) - q(x|M, \text{ACC}^1) \otimes q(y^j|M, \text{ACC}^j) \right\| \leq 3\frac{\epsilon_{deF}}{\xi^2}. \qquad (181)$$

□

Now we will combine Supplementary Proposition 23 which gives that from device 1 and device 2 we obtain probability distributions with upper bounded maximal probability and Supplementary Proposition 24 which says that the joint probability distribution is close to a product distribution.

Theorem 25. *Suppose we are given $\epsilon > 0$. Set $\delta > 0$ such that*

$$\frac{1}{3}\left(1 + 2\frac{\sqrt{2\delta}}{(\frac{1}{2} - \epsilon)^4}\right) < 1 \qquad (182)$$

(see Supplementary Figure 6 trade-off between δ and ϵ). Then for arbitrary family of probability distributions $p_w(x, y, z, u, v, t, j, e)$ satisfying conditions (144)-(151) there exists an extractor $s(x, y^j, t)$ with $|S| = 2^{n^c}$ values for a constant $c > 0$ (depending on ϵ) such that

$$d^{II}_{comp} \cdot p(ACC) \leq 2^{-n^{\Omega(1)}}. \qquad (183)$$

where d^{II}_{comp} is given by Eq. (140).

Remark 26. *Note, that due to* (144)*,* $p_w(ACC)$ *does not depend on* w*, hence we wrote just* $p(ACC)$ *in the theorem. Moreover, we even have* $q(ACC) = p(ACC)$*.*

Proof. Set $M = (z, u, v^j, M_j, e)$ and the probability distribution q given by (152). Let us consider $d_{\rm II}$, as in Eq. (158),

$$\begin{aligned} d_{\rm II} &= \sum_M q(M|\mathrm{ACC}) \sum_s \left| q(s|M,\mathrm{ACC}) - \frac{1}{|S|} \right| \\ &= \sum_{M \notin D_{\epsilon_{deF}}} q(M|\mathrm{ACC}) \sum_s \left| q(s|M,\mathrm{ACC}) - \frac{1}{|S|} \right| + \sum_{M \in S^\xi \cap D_{\epsilon_{deF}}} q(M|\mathrm{ACC}) \sum_s \left| q(s|M,\mathrm{ACC}) - \frac{1}{|S|} \right| \\ &+ \sum_{M \in (S^\xi)^c \cap D_{\epsilon_{deF}}} q(M|\mathrm{ACC}) \sum_s \left| q(s|M,\mathrm{ACC}) - \frac{1}{|S|} \right| \end{aligned} \tag{184}$$

We consider the three terms separately. Let us consider the first term with $M \notin D_{\epsilon_{deF}}$. By Eq. (50) from Supplementary Lemma 11, we know that

$$\mathbb{E}_{M \sim q(M)} \| q(x, y^j|M) - q(x|M) \otimes q(y^j|M) \| \leq \epsilon_{deF}^2, \tag{185}$$

where $\epsilon_{deF}^2 = c\sqrt{n} N^{\frac{1}{2}\log(\frac{1}{2}+\epsilon)}$ and c is an absolute constant. Applying Markov inequality to the above, we get that

$$\Pr_{q(M)} \left(\| q(x, y^j|M) - q(x|M) \otimes q(y^j|M) \| \leq \epsilon_{deF} \right) \geq 1 - \epsilon_{deF}, \tag{186}$$

so that $q(D_{\epsilon_{deF}}) \geq 1 - \epsilon_{deF}$. We therefore have, using $\sum_s \left| q(s|M,\mathrm{ACC}) - \frac{1}{|S|} \right| \leq 2$, that

$$\begin{aligned} \sum_{M \notin D_{\epsilon_{deF}}} q(M|\mathrm{ACC}) \sum_s \left| q(s|M,\mathrm{ACC}) - \frac{1}{|S|} \right| &\leq 2 \sum_{M \notin D_{\epsilon_{deF}}} \frac{q(M,\mathrm{ACC})}{q(\mathrm{ACC})} \\ &\leq 2 \sum_{M \notin D_{\epsilon_{deF}}} \frac{q(M)}{q(\mathrm{ACC})} \leq \frac{2\epsilon_{deF}}{q(\mathrm{ACC})}. \end{aligned} \tag{187}$$

Consider now the second term with $M \in S^\xi \cap D_{\epsilon_{deF}}$. Supplementary Proposition 23 implies that with probability

$$1 - \sqrt{\frac{\gamma^{\mu n} + 2\epsilon_{Az}}{q(\mathrm{ACC}_1)}} - \sqrt{\frac{\gamma^{\mu n} + 2\epsilon_{Az}}{q(\mathrm{ACC}_2)}} \geq 1 - 2\sqrt{\frac{\gamma^{\mu n} + 2\epsilon_{Az}}{q(\mathrm{ACC})}} \tag{188}$$

we have that for $h := \frac{1}{2}\left[\log(\gamma^{\mu n} + 2\epsilon_{Az}) + \log q(\mathrm{ACC})\right]$,

$$H_{\min}(q(x|M, \mathrm{ACC}^1)) \geq h \;\text{ and }\; H_{\min}(q(y^j|M, \mathrm{ACC}^j)) \geq h. \tag{189}$$

Moreover, by condition (156) and (154) (i.e. $q(t|x, y, z, u, v, j, e) = q(t|z, u, v, j, e)$), we obtain that $q(t|M, \mathrm{ACC})$ also satisfies the SV source conditions, i.e., $H_{\min}(q(t|M, \mathrm{ACC})) \geq cn$ for some constant c depending on ε. By Supplementary Lemma 3 part (ii), there exists an extractor that extracts $\Theta(h)$ bits $s = s(x, y^j, t)$. Let us denote the action of extractor by Extr. The extractor acts on

$$q(x, y^j, t|M, \mathrm{ACC}) = q(t|M, \mathrm{ACC}) \otimes q(x, y^j|M, \mathrm{ACC}) \tag{190}$$

producing the output s with distribution $q(s|M,\text{ACC})$ and so

$$\text{Extr}\left(q(x,y^j,t|M,\text{ACC})\right) \equiv q(s|M,\text{ACC}). \tag{191}$$

The action of the extractor on an ideal product distribution produces an output with some distribution which we denote $\bar{q}(s|M)$

$$\text{Extr}\left(q(t|M,\text{ACC}) \otimes q(x|M,\text{ACC}^1) \otimes q(y^j|M,\text{ACC}^j)\right) \equiv \bar{q}(s|M) \tag{192}$$

From Supplementary Lemma 3 part (ii), we know that

$$\sum_s \left|\bar{q}(s|M) - \frac{1}{|S|}\right| \leq 2^{-h^{\Omega(1)}}, \tag{193}$$

with $|S| = 2^{\Theta(h)}$. Since $M \in D_{\epsilon_{deF}}$, by Supplementary Proposition 24 we have that for $M \in S^\xi$

$$\|q(x,y^j|M,\text{ACC}) - q(x|M,\text{ACC}^1) \otimes q(y^j|M,\text{ACC}^j)\| \leq \frac{3\epsilon_{deF}}{\xi^2}, \tag{194}$$

so that, using Eq. (190), we have

$$\|q(t,x,y^j|M,\text{ACC}) - q(t|M,\text{ACC}) \otimes q(x|M,\text{ACC}^1) \otimes q(y^j|M,\text{ACC}^j)\| \leq \frac{3\epsilon_{deF}}{\xi^2}. \tag{195}$$

Hence, according to monotonicity of trace norm and Eq. (192) we obtain

$$\|q(s|M,\text{ACC}) - \bar{q}(s|M)\| \leq \frac{3\epsilon_{deF}}{\xi^2} \tag{196}$$

Thus, by using (193) and (196) and applying triangle inequality we get

$$\sum_s \left|q(s|M,\text{ACC}) - \frac{1}{|S|}\right| \leq 2^{-h^{\Omega(1)}} + \frac{3\epsilon_{deF}}{\xi^2} \tag{197}$$

and therefore the second term in Eq. (184) is estimated as follows

$$\sum_{M\in S^\xi\cap D_{\epsilon_{deF}}} q(M|\text{ACC}) \sum_s \left|q(s|M,\text{ACC}) - \frac{1}{|S|}\right| \leq 2^{-h^{\Omega(1)}} + \frac{3\epsilon_{deF}}{\xi^2}. \tag{198}$$

Now, we deal with the third term, i.e. with $M \in (S^\xi)^c \cap D_{\epsilon_{deF}}$. For $M \in (S^\xi)^c$ we have that $q(\text{ACC}|M) \leq \xi$. Using $\sum_s \left|q(s|M,\text{ACC}) - \frac{1}{|S|}\right| \leq 2$, we obtain

$$\begin{aligned}\sum_{M\in(S^\xi)^c\cap D_{\epsilon_{deF}}} q(M|\text{ACC}) \sum_s \left|q(s|M\text{ACC}) - \frac{1}{|S|}\right| &\leq 2 \sum_{M\in(S^\xi)^c\cap D_{\epsilon_{deF}}} q(M|\text{ACC}) \\ &\leq 2 \sum_{M\in(S^\xi)^c\cap D_{\epsilon_{deF}}} \frac{q(M)q(\text{ACC}|M)}{q(\text{ACC})} \leq \frac{2\xi}{q(\text{ACC})}\end{aligned} \tag{199}$$

Inserting (187), (199) and (198) into (184), we obtain

$$d_{\text{II}} \leq \frac{2\epsilon_{deF}}{q(\text{ACC})} + 2^{-h^{\Omega(1)}} + \frac{3\epsilon_{deF}}{\xi^2} + \frac{2\xi}{q(\text{ACC})}, \tag{200}$$

where ξ is an arbitrary number satisfying $\xi \geq 2\epsilon_{deF}$ (as in Supplementary Proposition 24), and $h = \frac{1}{2}\left[\log(\gamma^{\mu n} + 2\epsilon_{Az}) + \log q(\mathrm{ACC})\right]$. We now analyze the product $d_{\mathrm{II}} \cdot p(\mathrm{ACC})$.

We set $\delta_e = n^{-\frac{1}{4}}$, so that $\epsilon_{Az} = 2e^{-\frac{1}{4}\delta_e^2 n} = 2^{-\Omega(\sqrt{n})}$. We consider only $n \geq n_0$ where n_0 is such that $\delta_e \leq \delta$ (i.e. $n_0 = \lfloor \frac{1}{\delta^4} \rfloor$). Then $\mu \geq 1 - \sqrt{2\delta}$ and $\gamma < 1$. Now, let $\eta = \sqrt{\gamma^{\mu n} + 2\epsilon_{Az}}$, so that $\eta = 2^{-\Omega(\sqrt{n})}$. Suppose first that $q(\mathrm{ACC}) \leq \eta$ then since $d_{\mathrm{II}} \leq 2$, we have $d_{\mathrm{II}} \cdot q(\mathrm{ACC}) \leq 2\eta$. Suppose now, that $q(\mathrm{ACC}) \geq \eta$. Then setting $\xi = \eta^2$, we get $2^h = 2^{\Omega(\sqrt{n})}$ and, using (200), we obtain

$$d_{\mathrm{II}} \leq 2^{-n^{\Omega(1)}} + \frac{5\epsilon_{deF}}{\eta^4} + 2\eta. \tag{201}$$

Finally, recall that, due to Supplementary Lemma 11, $\epsilon_{deF}^2 = c\sqrt{n}N^{\frac{1}{2}\log(\frac{1}{2}+\epsilon)}$ where c is absolute constant. Setting the number of blocks $N = 2^n$ so that $\epsilon_{deF}^2 = 2^{-\Omega(n)}$, we obtain

$$d_{\mathrm{II}} \leq 2^{-n^{\Omega(1)}}. \tag{202}$$

so that

$$d_{\mathrm{II}} \cdot q(\mathrm{ACC}) \leq 2^{-n^{\Omega(1)}}. \tag{203}$$

By definition of q and Supplementary Remark 26, we have $q(\mathrm{ACC}) = p(\mathrm{ACC})$ and from Supplementary Proposition 21 we know that $d_{\mathrm{comp}}^{\mathrm{II}} \leq |S| d_{\mathrm{II}}$. We then see that there exists some constant $c > 0$ such that setting $|S| = 2^{n^c}$, we get $d_{\mathrm{comp}}^{\mathrm{II}} \cdot p(\mathrm{ACC}) \leq 2^{-n^{\Omega(1)}}$. This completes the proof. □

SUPPLEMENTARY NOTE 7: Conclusions and open questions

We have presented a protocol for obtaining secure random bits from an arbitrarily (but not fully deterministic) Santha-Vazirani source. The protocol uses a finite number (as few as four for the Bell inequality considered here) of no-signaling devices, and works even with correlations attainable by noisy quantum mechanical resources. Moreover, the correctness of the protocol is not based on quantum mechanics and only requires the no-signaling principle.

We leave the following open questions to future research:

- Is there an efficient protocol for device-independent randomness amplification with a constant number of devices, and tolerating a constant rate of noise, whose correctness only assumes limited independence between the source and the devices?
- Do Protocol I and II work even for a public SV source (in which the bits are drawn from the source and communicated to all the parties)?
- Is there a protocol that can tolerate a higher level of noise? What if we assume the validity of quantum mechanics?
- Can we amplify randomness with only a finite number of devices from other types of weak sources? A particularly interesting case is the min-entropy source [3, 22, 23].
- A more technical question is to improve the de Finetti theorem given in [8, 13]. What are the limits of de Finetti type results when the subsystems are selected from a Santha-Vazirani source?

- Finally suppose one would like to realize device-independent quantum key distribution with only an imperfect SV source as the randomness source. Is there an efficient protocol for this task tolerating a constant rate of noise and giving a constant rate of key? Here the question is open for both quantum-mechanical and no-signaling adversaries.

---

[1] R. Colbeck and R. Renner, Free randomness can be amplified, Nature Physics 8, 450–453 (2012).

[2] R. Gallego, L. Masanes, G. de la Torre, C. Dhara, L. Aolita and A. Acin, Full randomness from arbitrarily deterministic events, Nature Communications 4, 2654 (2013).

[3] K.M. Chung, Y. Shi and X. Wu, Physical randomness extractors: generating random numbers with minimal assumptions, Preprint at http://arxiv.org/abs/1402.4797 (2014).

[4] P. Mironowicz, R. Gallego and M. Pawłowski, Amplification of arbitrarily weak randomness. Phys. Rev. A 91, 032317 (2015).

[5] J. Bouda, M. Pawłowski, M. Pivoluska and M. Plesch, Device-independent randomness extraction for arbitrarily weak min-entropy source, Phys. Rev. A 90, 032313 (2014).

[6] M. Coudron, H. Yuen, Infinite randomness expansion with a constant number of devices, Proceedings of the 46th Annual ACM Symposium on Theory of Computing (STOC '14), 427–436 (2014).

[7] E. Chattopadhyay and D. Zuckerman, Explicit two-source extractors and resilient functions, Electronic colloquium on computational complexity, Revision 1 of Report No. 119 (2015).Functions. Electronic colloquium on computational complexity. Revision 1 of Report No. 119 (2015).

[8] F.G.S.L. Brandão and A.W. Harrow, Quantum de Finetti theorems under local measurements with applications, Proceedings of the 45th annual ACM symposium on Theory of computing (STOC'13), 861–870 (2013).

[9] F.G.S.L. Brandão, R. Ramanathan, A. Grudka, K. Horodecki, M. Horodecki, P. Horodecki, T. Szarek and H. Wojewódka, Robust device-independent randomness amplification with few devices, Preprint at http://arxiv.org/abs/1310.4544 (2015).

[10] S. Pironio, A. Acin, S. Massar, A. Boyer de la Giroday, D.N. Matsukevich, P. Maunz, S. Olmschenk, D. Hayes, L. Luo, T.A. Manning and C. Monroe, Random numbers certified by Bell's theorem, Nature 464, 1021–1024 (2010).

[11] S. Pironio and S. Massar, Security of practical private randomness generation, Phys. Rev. A 87, 012336 (2013).

[12] S. Fehr, R. Gelles and C. Schaffner, Security and composability of randomness expansion from Bell inequalities, Phys. Rev. A 87, 012335 (2013).

[13] F.G.S.L. Brandão and A.W. Harrow, Product-state approximations to quantum ground states, Proceedings of the forty-fifth annual ACM symposium on Theory of computing (STOC'13), 871–880 (2013).

[14] Xin-Li. Xin-Li, Extractors for a constant number of independent sources with polylogarithmic min-entropy, IEEE 54th Annual Symposium on Foundations of Computer Science (FOCS), 100–109 (2013).

[15] B. Chor and O. Goldreich, Unbiased bits from sources of weak randomness and probabilistic communication complexity, IEEE 26th Annual Symposium on Foundations of Computer Science, 429–442 (1985).

[16] A. Rao, Extractors for a constant number of polynomially small min-entropy independent sources, Proceedings of the 38th Annual ACM Symposium on Theory of Computing (STOC'06), 497–506 (2006).

[17] O. Gühne, G. Tóth, P. Hyllus, Philipp and H. Briegel, Bell Inequalities for Graph States, Phys. Rev. Lett. 95, 120405 (2005). Phys. Rev. Lett. **95**, 120405 (2005).

[18] M. Ben-Or, M. Horodecki, D.W. Leung, D. Mayers and J. Oppenheim, The universal composable security of quantum key distribution, Proc. of TCC 2005, LNCS, Springer, vol. 3378, 386–406 (2005).

[19] R. Renner and R. Köenig, Universally composable privacy amplification against quantum adversaries, Proc. of TCC 2005, LNCS, Springer, vol. 3378, 407–425 (2005).

[20] R. Arnon-Friedman, A. Ta-Shma, Limits of privacy amplification against nonsignaling memory attacks, Phys. Rev. A 86, 062333 (2012).

[21] M. Berta, O. Fawzi, V.B. Scholz, Quantum-proof randomness extractors via operator space theory, Preprint at http://arxiv.org/abs/1409.3563 (2015).

[22] M. Plesch and M. Pivoluska, Device-independent randomness amplification with a single device, Physics Letters A, Volume 378, Issue 40, 2938-2944 (2014).
[23] L.P. Thinh, L. Sheridan and V. Scarani, Bell tests with min-entropy sources, Phys. Rev. A 87, 062121 (2013).
